# Adiponectin Limits IFN-γ and IL-17 Producing CD4 T Cells in Obesity by Restraining Cell Intrinsic Glycolysis

**DOI:** 10.3389/fimmu.2019.02555

**Published:** 2019-10-29

**Authors:** Jayagopi Surendar, Stefan J. Frohberger, Indulekha Karunakaran, Vanessa Schmitt, Wiebke Stamminger, Anna-Lena Neumann, Christoph Wilhelm, Achim Hoerauf, Marc P. Hübner

**Affiliations:** ^1^Institute for Medical Microbiology, Immunology and Parasitology, University Hospital Bonn, Bonn, Germany; ^2^Unit for Immunopathology, Institute of Clinical Chemistry and Clinical Pharmacology, University Hospital Bonn, Bonn, Germany; ^3^German Center for Infection Research (DZIF), Partner Site Bonn-Cologne, Bonn, Germany

**Keywords:** adiponectin, obesity, T cells, inflammation, filariasis, helminth, glycolysis, adipose tissue

## Abstract

Compared to the innate immune system, the contribution of the adaptive immune response during obesity and insulin resistance is still not completely understood. Here we demonstrate that high fat diet (HFD) increases the frequencies of activated CD4+ and CD8+ T cells and frequencies of T cells positive for IFN-γ and IL-17 in the adipose tissue. The adipocyte-derived soluble factor adiponectin reduces IFN-γ and IL-17 positive CD4+ T cells from HFD mice and dampens the differentiation of naïve T cells into Th1 cells and Th17 cells. Adiponectin reduces Th17 cell differentiation and restrains glycolysis in an AMPK dependent fashion. Treatment with adult worm extracts of the rodent filarial nematode *Litomosoides sigmodontis* (LsAg) reduces adipose tissue Th1 and Th17 cell frequencies during HFD and increases adiponectin levels. Stimulation of T cells in the presence of adipocyte-conditioned media (ACM) from LsAg-treated mice reduces Th1 and Th17 frequencies and this effect was abolished when ACM was treated with an adiponectin neutralizing antibody. Collectively, these data reveal a novel role of adiponectin in controlling pro-inflammatory CD4+ T cells during obesity and suggest that the beneficial role of helminth infections and helminth-derived products on obesity and insulin resistance may be in part mediated by adiponectin.

**Graphical Abstract:**
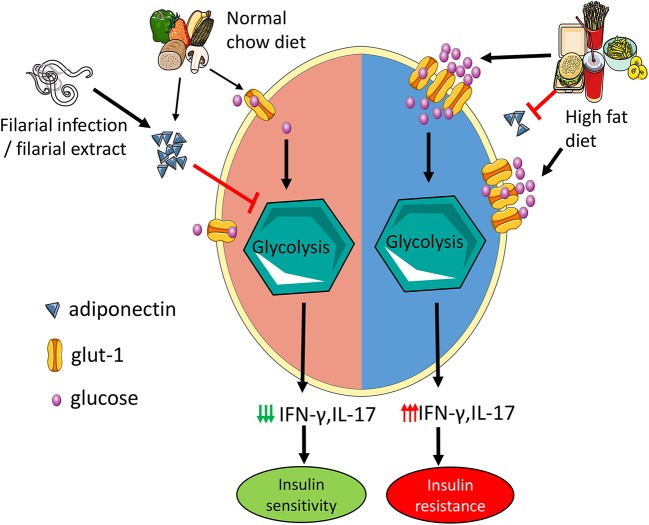
Adiponectin mitigates CD4 T cell inflammation by restraining glycolysis. Increased levels of adiponectin during normal chow diet limit Th1 and Th17 cell glycolysis which reduces the secretion of IFN-γ and IL-17 and results in improved insulin sensitivity. In high fat diet mice, hyperglycemia with concomitant reduction in adiponectin increases T cell glycolysis leading to increased IFN-γ and IL-17 production and insulin resistance. Administration of filarial extracts and assumedly filarial infection increase adiponectin levels, reduce IFN-γ and IL-17 production by T cells and improve insulin sensitivity.

## Introduction

With 2 billion overweight or obese individuals reported globally in 2013, the incidence of obesity is escalating at an alarming rate (1). There is a growing body of evidence linking innate immune cells in general and macrophages in particular to inflammation and insulin resistance (2). During obesity, macrophages infiltrate the expanding adipose tissue and switch from a predominant anti-inflammatory M2 phenotype to a pro-inflammatory M1 phenotype (3, 4). Recent reports have identified T cells as key players of the adaptive immune system in orchestrating the inflammatory effects of macrophages and thereby promoting insulin resistance (5, 6).

During obesity, increased numbers of memory T cells occur in the adipose tissue (7) and depletion of T cells in general or specifically from the adipose tissue has been shown to decrease adipose tissue inflammation in obese mice and to improve insulin resistance (7, 8). In addition to conventional antigen presenting cells like macrophages, dendritic cells, and B cells, non-immune cells such as adipocytes express antigen presenting molecules and together with adipocytokines, potentially regulate T cell activation in the adipose tissue (9, 10). However, there is no clear understanding of how T cell immune responses are modulated during obesity and how conventional and non-conventional antigen presenting cells modulate T cell inflammation.

Soluble factors secreted by adipocytes such as lipids and adipocytokines including leptin or adiponectin and their downstream signaling events can modulate T cells (10, 11). Another facet of adipocytokine-mediated regulation of T cell function is their influence on the nutritional demands of these cells that in turn tightly control their metabolic function. Effector T cell function is fuelled by glucose through aerobic glycolysis which is increasingly available in insulin resistance (12). Leptin has been recently shown to be a crucial factor for the maintenance of the metabolic effects of activated T cells (13) and fasting-induced hypoleptinemia decreased IFN-γ and IL-17 production and the expression of a key glycolytic enzyme in Th17 cells during experimental autoimmune encephalomyelitis (14). Although adiponectin was shown to regulate T cell activity by modulating dendritic cell functions (10), it is not clear yet, if adiponectin regulates T cell activity directly.

In the recent past, the hygiene hypothesis has been extended from allergic to metabolic diseases such as obesity and diabetes (15), as several human studies have shown an inverse association between helminth infection and diabetes (16, 17). Similarly, experimental animal studies proved that helminth infection or helminth-derived products improve metabolic disorders of obesity (18, 19). Most of the studies have shown that helminth infection or helminth-derived products skew the adipose tissue immune environment toward an immunoregulatory phenotype with an expansion of M2 macrophages, eosinophils, regulatory T cells, and type 2 innate lymphocytes (ILC2s), which may alleviate adipose tissue inflammation (15, 20). We have reported that infection with the rodent filarial nematode *Litomosoides sigmodontis* or administration of crude *L. sigmodontis* adult worm extract (LsAg) improve glucose tolerance in obese mice (19). In the present study, we demonstrate that treatment with LsAg modulates CD4+ T cell activation during obesity via an adiponectin mediated mechanism and provide evidence for the role of the potential insulin sensitizing adipokine adiponectin in regulating T cell function by restraining Th1 and Th17 glycolysis during high fat diet (HFD).

## Materials and Methods

### Ethics Statement

Animal housing conditions and the procedures used in this work were performed according to the European Union animal welfare guidelines. All protocols were approved by the Landesamt für Natur, Umwelt und Verbraucherschutz, Cologne, Germany (84-02.04.2016.A331).

### Mice

All mice were maintained in ventilated cages with a 12-h day/night cycle, food and water *ad libitum*. The experiments were carried out with male C57BL/6J mice purchased from Janvier Labs (Le Genest-St.-Isle, France). Mice were maintained at the animal facilities of the University Hospital Bonn and were fed with either normal chow diet (NCD) (15% fat) or HFD (caloric information: 60% kilocalories fat; 20% carbohydrate and 20% protein; Research Diets, Inc., Brogaarden, Denmark). Six week-old male mice were fed with HFD for 12–16 weeks and control mice received NCD.

### Glucose Tolerance and Insulin Tolerance Test

After 6 h fasting, the glucose tolerance test (GTT) was performed. Mice were intraperitoneally (i.p.) administered with 1 g of glucose/kg of body weight. Blood glucose levels were measured from tail vein blood at 0, 30, 60, and 120 min after glucose administration using a blood glucometer (AccuCheck Advantage; Roche Diagnostics GmbH, Mannheim, Germany). The insulin tolerance test (ITT) was carried out 4 h after fasting. One Unit of insulin/kg body weight of human insulin (Sanofi-Aventis, Frankfurt, Germany) was i.p. injected and blood glucose levels were measured at 0, 30, 60, and 120 min after insulin administration. The area under the curve (AUC) was obtained by calculating the area between the x-axis and a given curve using GraphPad Prism software (version 5.03; GraphPad Software, San Diego, Calif., USA).

### Stromal Vascular Fraction and Splenocytes Isolation

Twelve to sixteen weeks after the HFD, blood was taken from control and HFD fed mice by piercing the vena facialis with lancets (Goldenrod, Braintree Scientific, Braintree MA, USA). The blood was directly transferred to EDTA tubes. The blood was centrifuged at room temperature and plasma was stored at −80°C until use.

Subsequently, mice were euthanized by an overdose inhalation of isoflurane (Forene®, Abbolt, Wiesbaden, Germany). After dissecting the skin and peritoneum of the euthanized mice, epididymal fat was excised and kept in ice cold DMEM (Gibco, Thermo Fischer Scientific; Darmstadt, Germany) media. The spleen was surgically removed and kept in ice cold RPMI-1640 medium (Gibco)

Stromal vascular fraction (SVF) was isolated from NCD and HFD mice as described elsewhere (8). In brief, the epididymal fat pad was excised from the male mice, minced, and digested with 0.2 mg/ml of collagenase (Sigma-Aldrich; Taufkirchen, Germany) containing DMEM medium for 40 min at 37°C with constant shaking. The floating adipocytes were removed and the SVF pellet was filtered through a 40 μm filter after red blood cell lysis (Invitrogen, Thermo Fischer scientific).

Single cell suspension from spleen was prepared by mechanically forcing splenocytes through a 70 μm cell filter (BD Biosciences; Heidelberg, Germany) using syringe plungers. Red blood cells were then lysed using ACK Lysing Buffer (Thermo Fischer Scientific) (21). The cells were used for culture and flow cytometry after cell counts were obtained.

### Cell Culture

After the enumeration of the cell number from the SVF and splenocyte single cell suspension, cells were cultured in 12-well tissue culture plates at concentrations of 1 × 10^6^/ml in the presence of phorbol myristate acetate (PMA) (50 ng/ml) and ionomycin (1 μg/ml) for 6 h in RPMI-1640 medium (Gibco) at 37°C. After 2 h, Golgi Stop/Golgi Plug (BD Biosciences) was added 4 h before harvesting the cells.

CD4+ T cells were isolated from NCD or HFD mice splenocytes using microbeads according to the manufacturer's protocol (Miltenyi Biotec; Bergisch Gladbach, Germany). In brief, single cell suspension of splenocytes were incubated with 10 μl of CD4 microbeads for 10 min at 4°C and CD4+ T cells were sorted using the autoMACS Pro Separator. The negative fraction was incubated with CD8+ microbeads and CD8+ T cells were sorted.

Isolated splenic CD4+ T cells from NCD or HFD mice were cultured with adipocytokines and fatty acids in previously reported concentrations shown below. T cells were stimulated with anti-CD3 (5 μg/ml) and anti-CD28 (2 μg/ml) in the presence of adiponectin (5 μg/ml) (Peprotech; Hamburg, Germany) (22), leptin (250 ng/ml) (Peprotech) (13, 23), palmitic acid (Sigma-Aldrich) (0.5 mM) (24) and oleic acid (100 μM) (Sigma-Aldrich) (25).

### Adipocytes—T Cell Co-culture

Adipocyte isolation was done as previously described (26). Briefly, 100 mg of adipose tissue was digested with collagenase, the adipocytes were washed thrice with fresh media. The adipocytes from NCD or HFD mice were cultured with purified splenic CD4+ T cells or CD8+ T cells at concentrations of 0.5 × 10^6^/ml (Miltenyi Biotec) from NCD or HFD mice as previously described (27) in the presence of anti-CD3 and anti-CD28 as well as Golgi Stop/Golgi Plug (BD Biosciences) for 4 h. Transwell experiments were performed by placing NCD or HFD adipocytes in the upper chamber and CD4+ T cells in the lower chamber. Control wells had only T cells without adipocytes. T cells were treated with anti-CD3 and anti-CD28 and Golgi Stop/Golgi Plug.

### Macrophages and B Cell Depletion

During the first week of the HFD, macrophages and B cells were depleted as described elsewhere (28, 29). Macrophages were depleted by i.p. injection of 150 μl of clodronate liposomes (Clodronate Liposomes Foundation; The Netherlands; http://clodronate.liposomes.com) and the control mice received equal volumes of PBS liposomes. Three days after the initial injection, the second dose was given and the SVF isolation was performed 16 week after the start of HFD. B cell depletion was performed using anti-mouse mCD20 antibody (Biogen; clone 18B12, isotype IgG2a). 250 μg of antibody was injected followed by a second injection 4 days later during the early phase of the HFD.

### Western Blot

CD4+ T cells from NCD and HFD mice were cultured with anti-CD3 and anti-CD28 for 15 min and the cells were lysed in RIPA buffer (Thermo Fisher Scientific) for protein extraction. Protein separation was done by SDS-PAGE and transferred onto a nitrocellulose membrane. The membrane was incubated with primary antibodies for pAMPK or β-actin (Cell Signaling Technology; Frankfurt, Germany) overnight. Then the membrane was incubated with an HRP-conjugated secondary antibody (Cell Signaling Technology) for 1 h. Pierce™ ECL Western Blotting Substrate (Thermo Fisher Scientific), VersaDoc 5000 imaging system (Bio-Rad; Hercules, CA, USA), and Image J program were used for detection, visualizing the membranes and quantification, respectively.

### T Cell Purification

Twelve to 16 weeks after HFD or NCD, splenic CD4+ T cells were isolated by magnetic cell separation (MACS) using the Miltenyi kit. The purified CD4+ T cells were incubated with Fc-block (Thermo Fisher Scientific) followed by staining with CD4-PE-Cy7, CXCR3-FITC, CCR4-PE, and CCR6-APC for 30 min in the dark. All antibodies were obtained from Biolegend (Fell, Germany). Dead cells were excluded by DAPI (BD Biosciences) staining and Th1 cells (CD4+CXCR3+CCR6–) and Th17 cells (CD4+CCR4+CCR6+CXCR3–) were purified using the BD FACS Aria III high-speed cell sorter (BD Biosciences).

### Real-Time PCR

Sorted Th1 and Th17 cells from NCD and HFD mice were stored in 350 μl RLT buffer (Qiagen; Hilden, Germany) at −80°C. Total RNA was extracted from the purified Th1 and Th17 cells using the RNeasy mini kit (Qiagen). Total RNA was reverse transcribed with the Omniscript RT Kit (Qiagen) according to the manufacturer's instructions with oligo-d(T) primers (Roche; Penzberg, Germany). Real-time PCR was performed with the Thermo Fisher QuantStudio 5 using the TaqMan universal PCR master mix (Thermo Fischer Scientific). TaqMan probes for hexokinase 1(*hk1*), pyruvate kinase (*pkm*), lactate dehydrogenase (*ldh*), glucose transporter-1 (*glut-1*), adiponectin receptor-1 (*adipor1*) were analyzed and hypoxanthine-guanine phosphoribosyltransferase (*hprt*) was used as an endogenous control (Thermo Fisher Scientific). The relative CT (threshold cycle at the exponential phase of amplification) method was used to calculate the qPCR results. Delta CT was calculated as CT (gene of interest)—CT (*hprt*). The fold change was calculated as 2^−Δ*CT*^ as previously described (30).

### Th1 and Th17 Cell Differentiation

Splenic naive CD4+ T cells (CD4+CD62L+CD44–) from HFD mice were isolated according to the manufacturer's instructions (Miltenyi Biotec). Differentiation of naïve CD4+ T cells into Th1 and Th17 cells were performed as previously described with some modifications (31, 32). In brief, 48 well culture plates were coated with anti-CD3 (1 ug/ml) and anti-CD28 (5 ug/ml) in PBS and incubated for 3 h at 37°C. Purified naïve CD4+ T cells (0.5 × 10^6^ cells/well in 0.5 ml of RPMI) were differentiated into Th1 cells in the presence of IL-12 (Peprotech) and anti-mouse IL-4 (Peprotech) at the concentrations of 3 and 10 μg/mL, respectively, for 96 h in RPMI containing 10% FCS (Gibco). For Th17 cell differentiation, naïve T cells were incubated with IL-6 (Peprotech) and TGFβ1 (Peprotech) at 20 ng/ml and 1 ng/ml in complete RPMI media for 96 h.

### Seahorse Analysis

To analyse the extracellular acidification rate (ECAR; in mpH/min), the Seahorse XF^e^96 metabolic extracellular flux analyzer was used (Seahorse Bioscience; North Billerica, MA, USA). Differentiated Th1 and Th17 cells were cultured in XF media (Agilent; Ratingen, Germany) supplemented with 10% FCS and 10 mM glucose (Thermo Fischer Scientific) and analyzed with an XF-96 Extracellular Flux Analyzer. At least three consecutive measurements were recorded after the stimulation with anti-CD3/anti-CD28 followed by the addition of 5 μg/ml of adiponectin and 10 μM compound C (Merck Millipore, Darmstadt, Germany) (22) to inhibit AMPK signaling.

### LsAg Treatment

LsAg was prepared as described previously (33). In brief, *L. sigmodontis* adult worms were harvested from infected gerbils' thoracic cavities and mechanically homogenized on ice in endotoxin-free PBS (PAA; Pasching, Austria). The supernatant was collected and protein quantification was done by Bradford assay (Cytoskeleton; Denver, CO., USA). Aliquots of LsAg were stored for later usage at −80°C.

LsAg treatment was performed as previously described (19). Daily i.p. injections of 2 μg LsAg per mouse for 2 weeks were given to obese mice during weeks 14–16 of HFD. Corresponding control mice received PBS injections. After the final LsAg injection, the GTT and immunological studies were performed.

### Conditioned Media Culture

CD4+ T cells from spleens of obese mice were cultured in adipocyte-conditioned media from PBS or LsAg treated mice with PMA and ionomycin as described above. For adiponectin neutralization experiments, adipocyte conditioned media was treated overnight with 10 μg/ml of adiponectin-neutralizing antibody (anti-adiponectin, AF1119, anti-mAcrp30; R&D Systems, Minneapolis, MN., USA) or isotype control anti-goat IgG (10 μg/ml; R&D Systems) (34). This neutralized media was used to culture CD4+ T cells from HFD mice in the presence of PMA/ionomcycin.

### Insulin ELISA and HOMA-IR Calculation

Fasting plasma insulin was measured according to the manufacturer's protocol (Crystal Chem; IL, USA). Homeostasis model assessment of insulin resistance (HOMA-IR) was obtained by multiplying the fasting glucose [mmol/l] with the fasting insulin levels [μU/ml] followed by division by 22.5 (35).

### Adiponectin ELISA

Adipocytes were isolated from the same weight of adipose tissue from PBS and LsAg treated mice. The isolated adipocytes were cultured overnight and the adipocyte-conditioned media (ACM) was collected and stored at −80°C until use. Adiponectin levels were measured in the plasma and ACM by ELISA according to the manufacturer's instructions (R&D system; Wiesbaden-Nordenstadt, Germany).

### Flow Cytometry

For *ex vivo* staining, single cell suspensions from SVF and splenocytes were stained with fluorochrome-conjugated antibodies against the following surface antigens: CD4-PE-Cy7, CD8α-PerCP-Cy5.5, NK1.1-APC-Cy7, CD69-PE, F4/80-APC, CD19-APC, MHCII-FITC, CD86-PerCP-Cy5.5 for 30 min. For *in vitro* staining, after the stimulation, harvested cells were incubated overnight in fixation/permeabilization buffer (eBiosciences; Darmstadt, Germany), blocked with PBS/1% BSA including 0.1% rat IgG (Sigma-Aldrich). Cells were stained with fluorochrome-conjugated antibodies against any combination of the following surface antibodies, CD4, CD8, and intracellular antigens IFN-γ, IL-4, IL-5, IL-13, and IL-17A. For pS6 staining, CD4+ T cells from NCD and HFD mice were stimulated with anti-CD3/anti-CD28 for 30 min and cells were stained for the surface marker-CD4-PE-Cy7 for 30 min. After washing, the cells were incubated with fixation/permeabilization buffer (eBioscience) for 20 min. The cells were then incubated with ice cold 90% methanol for 30 min followed by staining for pS6 in permeabilization buffer. CD4-PE-Cy7 and pS6-PE antibodies were purchased from eBioscience and all the other antibodies were procured from Biolegend (Fell, Germany). Data were acquired using a BD FACS Canto I System (BD Biosciences Heidelberg, Germany). FACS data were analyzed with FlowJo (Flowjo LLC; Ashland, Oregon) software. During analysis, gates were set using the FMO (fluorescence minus one) approach.

### Statistics

Statistical analyses was performed with the GraphPad Prism software Version 5.03 (GraphPad Software, San Diego, CA, USA). Differences between two unpaired groups were tested for statistical significance with the Mann–Whitney-*U*-test. *P*-values of <0.05 were considered statistically significant.

## Results

### HFD Skews Adipose Tissue Toward a Pro-inflammatory Milieu

**Figure 1:**
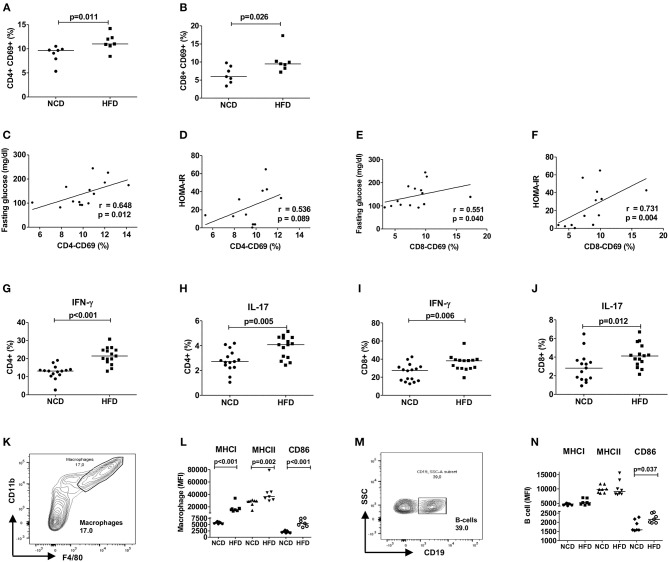
HFD induces an inflammatory milieu in the adipose tissue. **(A,B)** Frequency of CD69+CD4+ T cells **(A)** and CD69+CD8+ T cells **(B)** within the vascular adipose tissue T cells of mice fed with normal control diet (NCD) or 60% high fat diet (HFD) for 12–16 weeks (pooled data from *n* = 2 experiments, 3–4 mice each). **(C,E)** Spearman correlation between fasting plasma glucose and CD4+CD69+ T cells **(C)** and CD8+CD69+ T cells **(E)**. **(D,F)** Correlation between HOMA-IR and CD4+CD69+ T cells **(D)** and CD8+CD69+ T cells **(F)**. **(G–J)** Frequency of IFNγ + **(G,I)** and IL-17+ **(H,J)** CD4+ T cells and CD8+ T cells, respectively (pooled data from *n* = 3 experiments, 4–5 mice each). Cytokine expression of T cells was determined following PMA/Ionomycin stimulation. **(K,L)** Gating strategy for macrophages **(K)** and MFI of MHCI, MHCII, and CD86 on the gated macrophages **(L)**. **(M,N)** Gating scheme for B cells **(M)** and MFI of MHCI, MHCII, and CD86 on the gated B cells **(N)** (pooled data from *n* = 2–3 experiments, 3–4 mice each). **(A,B,G–J,L,N)** Two-tailed non-parametric Mann–Whitney *U*-test was performed.

To examine the role of T cells in mediating HFD-induced inflammation, we investigated the adipose tissue T cell activation and the secretion of pro-inflammatory cytokines from CD4+ and CD8+ T cells after 12–16 weeks of HFD. The study design is shown in Supplementary Figure 1A. As expected, HFD mice fed for 12–16 weeks on a HFD showed a higher body weight, adipose tissue weight as well as impaired glucose and insulin tolerance compared to NCD mice (Supplementary Figures 1B–G). The frequencies of activated CD69+ CD4+ (Figure 1A) and CD8+ T cells (Figure 1B) increased upon HFD and a positive correlation of the activation status of CD4+ and CD8+ T cells with fasting plasma glucose and HOMA-IR was found (Figures 1C–F). In addition, CD69+ CD4+ and CD8+ T cells showed a positive correlation with body weight (CD4+: *r* = 0.556, *p* = 0.038 and CD8+: *r* = 0.636, *p* = 0.026) and adipose tissue weight (CD4+: *r* = 0.648, *p* = 0.012 and CD8+: *r* = 0.565, *p* = 0.035). Further, frequencies of IFN-γ+ (Figure 1G) and IL-17+ CD4+ T cells (Figure 1H) and CD8+ T cells (Figures 1I,J) were increased in the adipose tissue of HFD mice compared to NCD fed mice. The gating strategy for T cells is shown in Supplementary Figure 2.

Similar to adipose tissue, splenic CD4+ T cells of obese mice showed higher expression of IFN-γ and IL-17 (Supplementary Figures 3A,B) and CD8+ T cells increased the expression of IFN-γ, but not IL-17 (Supplementary Figures 3C,D). As NK cells are also producers of type 1 and type 17 cytokines, the frequencies of NK cells positive for IFN-γ and IL-17 were also analyzed. In the adipose tissue, frequencies of IFN-γ+ and IL-17+ NK cells were significantly increased during HFD (Supplementary Figures 4A,B), whereas the frequencies of IFN-γ+ and IL-17+ NK cells in the spleen did not differ significantly between HFD and NCD mice (Supplementary Figures 4C,D). Th2 cells, which are involved in the resolution of inflammation, were also quantified based on their expression of IL-4+, IL-5+, and IL-13+ in the adipose tissue and spleen. HFD feeding increased the frequencies of IL-4+ CD4+ T cells in the adipose tissue, while no differences were observed in the frequencies of IL-5+ and IL-13+ CD4+ T cells in the adipose tissue and IL-4+, IL-5+, and IL-13+ splenic T cells of NCD and HFD mice (Supplementary Figures 4E–J). Systemic plasma concentrations of pro- (IL-1β, IL-6, and TNF) and anti-inflammatory cytokines (IL-10) were also evaluated, but were below the detection limit of the ELISA (data not shown). Together, these results confirm that HFD skews T cells toward a pro-inflammatory phenotype with increased levels of IFN-γ and IL-17 in the adipose tissue and partly in the spleen.

Next, we assessed if HFD feeding also affects antigen presentation on macrophages and B cells and thus the activation of T cells. In line with T cell inflammation in adipose tissue, obesity increased the expression of antigen presenting molecules MHCI and MHCII and co-stimulatory molecule CD86 on macrophages (Figures 1K,L) and CD86 on B cells (Figures 1M,N). CD4+ T cell activation showed a positive correlation with macrophage MHCII (*r* = 0.629, *p* = 0.016) and CD86 (*r* = 0.741, *p* = 0.002) expression and activated CD8+ T cells showed positive association with macrophage MHCI (*r* = 0.560, *p* = 0.037) and CD86 (*r* = 0.526, *p* = 0.056) expression. The expression of MHCI (*r* = 0.591, *p* = 0.026) and CD86 (*r* = 0.762, *p* = 0.001) on B cells showed also a statistically significant correlation with CD8+ T cell activation, but not CD4+ T cell activation. Collectively, these findings suggest that HFD feeding induces T cell activation, increases production of pro-inflammatory cytokines from CD4+ and CD8+ T cells and increases expression of antigen presenting molecules on macrophages and B cells.

### Macrophage and B Cell Depletion Improves CD8+ T Cell Inflammation

**Figure 2:**
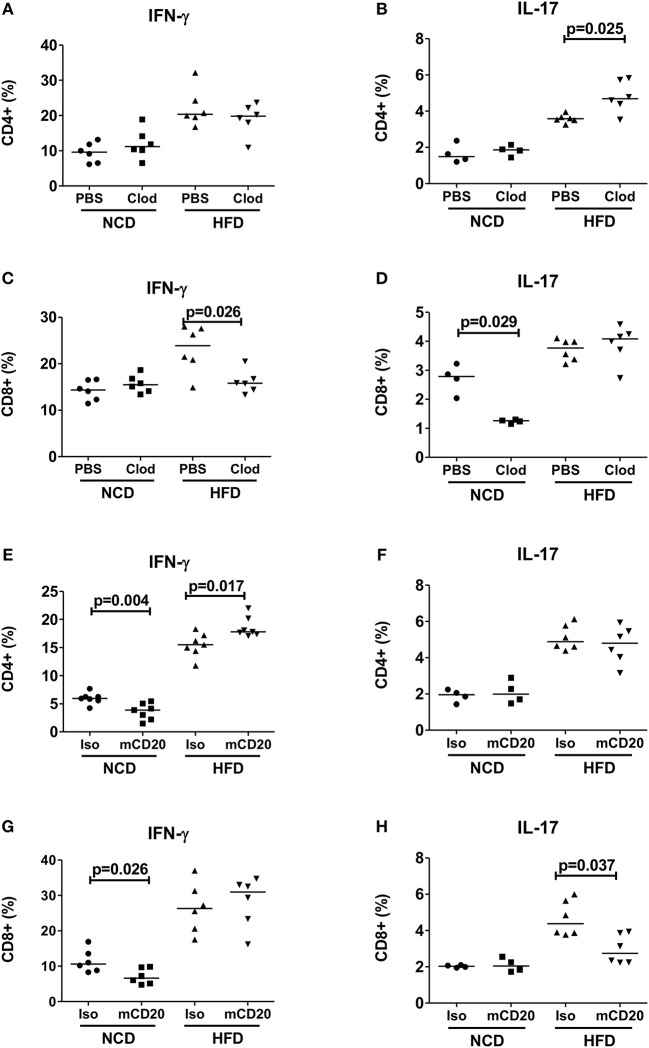
Depletion of macrophages or B cells reduces CD8+ T cell inflammation in obesity. **(A,B)** CD4+ IFN-γ **(A)** and IL-17 **(B)** positive cell frequencies in mice receiving clodronate and PBS liposome treatment with the onset of high fat diet (HFD) (pooled data from *n* = 2 experiments, three mice each) and NCD mice served as control. **(C,D)** Frequency of CD8+ IFN-γ **(C)** and IL-17 **(D)** positive cells in clodronate liposomes treated vs. control-liposome treated mice. **(E,F)** IFN-γ **(E)** and IL-17 **(F)** positive CD4+ T cell frequencies between mCD20 treated and isotype control treated mice. **(G,H)** IFN-γ **(G)** and IL-17 **(H)** positive CD8+ T cell frequencies in mCD20 treated and isotype antibody treated mice (pooled data from *n* = 2–3 experiments, 3–4 mice each). Two-tailed non-parametric Mann–Whitney *U*-test was performed to assess statistical significance between depleted and non-depleted HFD or NCD groups.

Based on our observation that increased expression of antigen presenting molecules on macrophages and B cells correlated with T cell inflammation, we next investigated if depletion of macrophages and B cells in obese mice could reduce the production of pro-inflammatory cytokines. Depletion of macrophages at the beginning of HFD onset, i.e., during the first week of HFD, did not affect frequencies of CD4+ IFN-γ+ (Figure 2A) cells, but significantly increased frequencies of IL-17 producing CD4+ T cells (Figure 2B, *p* = 0.025). In contrast, macrophage depletion resulted in a significant reduction of CD8+ IFN-γ+ (Figure 2C), but not CD8+ IL-17+ T cells (Figure 2D). In NCD mice, macrophage depletion had no effect on frequencies of CD4+ IFN-γ+ or CD4+ IL-17+ cells (Figures 2A,B), but the frequency of CD8+ IL-17+ cells was significantly reduced (Figure 2D).

On the other hand, B cell depletion during HFD resulted in a significant increase of CD4+ IFN-γ+ cells (Figure 2E), but had no effect on the frequencies of CD4+ IL-17+ and CD8+ IFN-γ+ T cells (Figures 2F–G), but significantly decreased IL-17+ CD8+ T cells (Figure 2H). B cell depletion reduced the frequencies of IFN-γ+ CD4+ and CD8+ T cells in NCD mice (Figures 2E,G). Taken together, ablation of macrophages and B cells during early stages of obesity decreases IFN-γ+ and IL-17+ CD8+ T cell frequencies, respectively. However, in case of CD4+ T cells, depletion of macrophages or B cells exacerbated the production of inflammatory cytokines.

### Adipocyte-Derived Factors Modulate CD4+ T Cell Function

**Figure 3:**
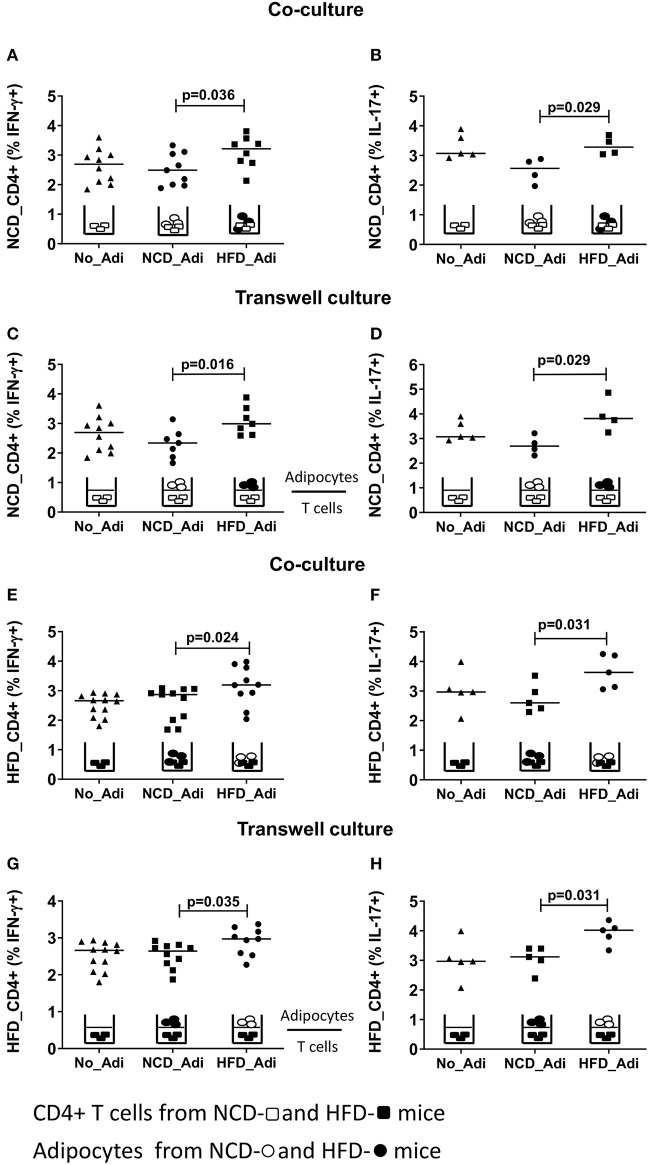
Adipocytes regulate CD4+ T cell cytokine production. Adipocytes from normal control diet (NCD) and high fat diet (HFD) mice were co-cultured or cultured in trans-wells with splenic CD4+ T cells of NCD and HFD mice in the presence of anti-CD3/CD28 and T cells without adipocytes served as controls. **(A,B)** Frequencies of CD4+ IFN-γ **(A)** and IL-17 **(B)** positive T cells from NCD mice after co-culture with no adipocytes (No_Adi), lean (NCD_Adi), or obese adipocytes (HFD_Adi) in the presence of anti-CD3/CD28. **(C,D)** Frequencies of IFN-γ **(C)** and IL-17 **(D)** positive NCD mice CD4+ T cells in adipocyte-T cell transwell culture. **(E,F)** IFN-γ **(E)** and IL-17 **(F)** CD4+ T cell frequencies from HFD mice after co-culture with lean or obese adipocytes in the presence of anti-CD3/CD28. **(G,H)** Frequencies of IFN-γ **(G)** and IL-17 **(H)** positive HFD mice CD4+ T cells in transwell culture with lean or obese adipocytes in the presence of anti-CD3/CD28 (Pooled data from *n* = 1–2 wells, three experiments). Two-tailed non-parametric Mann–Whitney *U*–test was performed to test for statistical significance between NCD_Adi and HFD_Adi groups.

Having established that conventional antigen presenting cells do not reduce CD4+ T cell inflammation during early HFD, we next tested if adipocytes, which are involved in extensive crosstalk with the immune cells, mediate these effects. Hence, splenic CD4+ T cells from NCD mice were co-cultured with adipocytes from HFD or NCD mice in the presence of anti-CD3/anti-CD28 and T cells cultured without adipocytes served as control. Adipocytes from obese mice increased the frequencies of IFN-γ+ (Figure 3A) and IL-17+ (Figure 3B) CD4+ T cells from NCD mice. This observation prompted us to test whether the effect of adipocytes on CD4+ T cells was contact dependent or mediated by soluble factors. Obese adipocytes increased IFN-γ+ and IL-17+ CD4+ T cell frequencies from NCD mice even in the absence of direct contact with the T cells in transwell plates (Figures 3C,D), suggesting that the effect of adipocytes on CD4+ T cells is mediated by soluble factors. In addition, co-culture and stimulation of HFD CD4+ T cells with anti-CD3/anti-CD28 in the presence of NCD adipocytes reduced IFN-γ+ and IL-17+ CD4+ T cell frequencies (Figures 3E,F), an effect likewise facilitated by adipocyte-derived factors (Figures 3G,H). However, stimulating NCD CD8+ T cells with HFD adipocytes (Supplementary Figures 5A,B) or HFD CD8+ T cells with NCD adipocytes (Supplementary Figures 5C,D) did not affect the cytokine positive T cell frequencies. These results indicate that adipocytes are indispensable for coordinating CD4+ T cell, but not CD8+ T cell inflammation in adipose tissue.

### Adiponectin Is a Crucial Factor for CD4+ T Cell Activation

**Figure 4:**
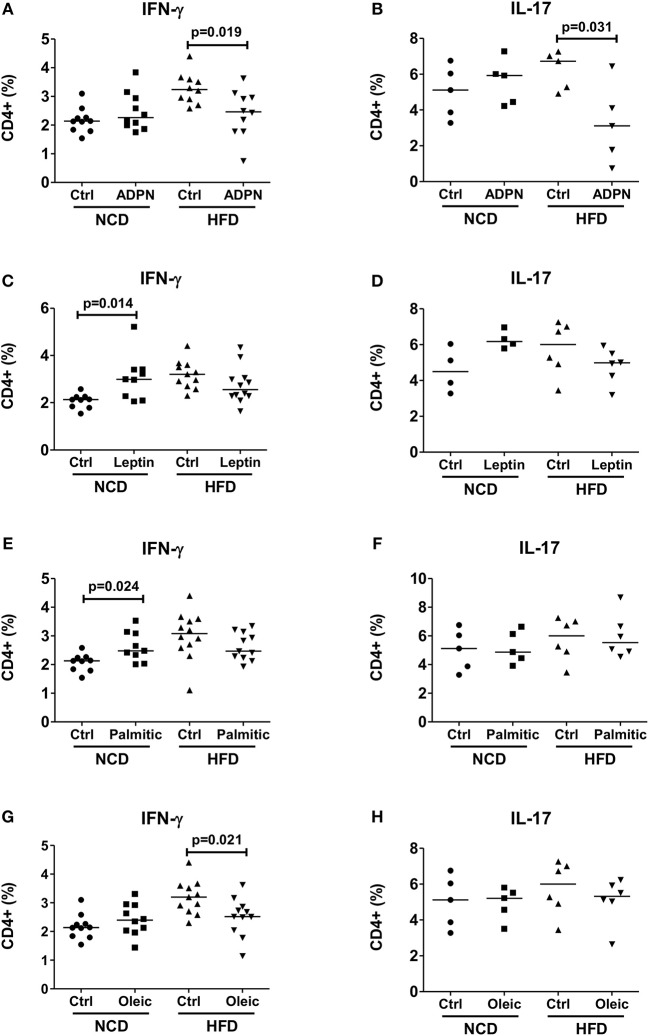
Adiponectin modulates T cell cytokine production from HFD mice. **(A–H)** CD4+ T cells were isolated from lean mice and cultured with fatty acids and adipocytokines in the presence of anti-CD3/CD28. Frequencies of IFN-γ and IL-17 positive CD4+ T cells upon anti-CD3/anti-CD28 stimulation in the presence of adiponectin (ADPN; **A,B**), leptin **(C,D)**, palmitic acid (Palmitic; **E,F**) and oleic acid (Oleic; **G,H**) (Pooled data from *n* = 1–2 wells, three experiments). Two-tailed non-parametric Mann–Whitney *U*-test was used to compare control and treated groups in NCD and HFD CD4+ T cells.

In order to delineate the adipocyte-derived molecule regulating T cell inflammation, we first targeted the prominent adipokines, adiponectin, and leptin, which have opposite effects on metabolism (36). Intriguingly, stimulating CD4+ T cells with anti-CD3/anti-CD28 in the presence of adiponectin reduced frequencies of IFN-γ+ and IL-17+ CD4+ T cells from HFD, but not from NCD CD4+ T cells (Figures 4A,B). Leptin, on the other hand, showed a moderate effect by increasing the IFN-γ+ CD4+ T cell but not IL-17+ CD4+ T cell frequencies from NCD mice (Figures 4C,D). The abundantly expressed fatty acids within the adipocyte fraction—palmitic acid and oleic acid were also tested. When CD4+ T cells from NCD and HFD mice were stimulated by TCR ligation (anti-CD3/anti-CD28) in the presence of palmitic acid or oleic acid, palmitic acid increased IFN-γ+ CD4+ T cell frequencies only from NCD mice (Figure 4E). However, presence of palmitic acid did not affect IL-17+ T cell frequencies either from NCD or HFD T cells (Figure 4F). While oleic acid reduced CD4+ IFN-γ+ T cell frequencies from HFD mice (Figure 4G), it had no effect on frequencies of IL-17+ CD4+ T cells (Figure 4H). Together, these results point to an important role for adiponectin in regulating CD4+ T cell inflammation in obesity.

### Adiponectin Inhibits Th17 Glycolysis in an AMPK Dependant Manner

**Figure 5:**
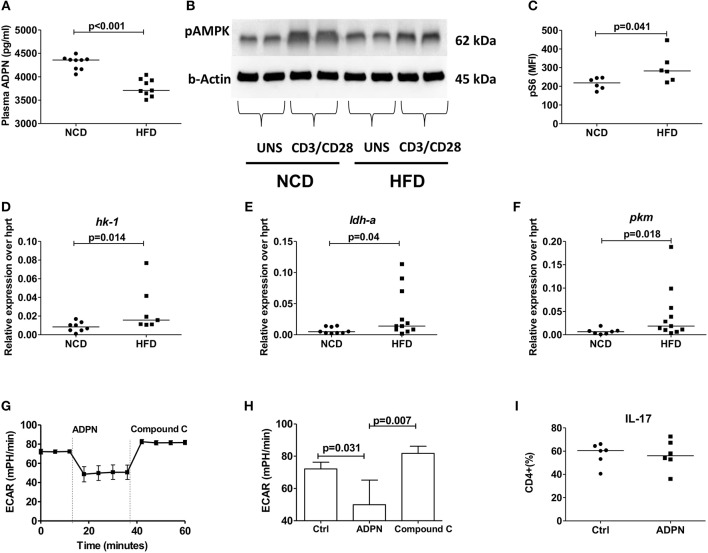
Obesity increases Th17 cell glycolysis and adiponectin dampens Th17- glycolysis in an AMPK dependent manner. **(A)** Plasma adiponectin levels in normal control diet (NCD) and high fat diet (HFD) fed mice. Western blot analysis of phospho-AMPK was determined. CD4+ T cells from NCD and HFD fed mice were stimulated with anti-CD3/CD28 for 15 min and expression of phospho-AMPK was measured **(B)**. **(C)** MFI of pS6 on CD4+ T cells in NCD and HFD mice following 30 min culture with anti-CD3/CD28. **(D–F)** Relative messenger RNA expression of glycolytic enzymes *hk1* **(D)**, *ldh-a* **(E)**, and *pkm* **(F)** in purified Th17 cells in NCD and HFD mice. **(G,H)** Extracellular acidification rate was measured by seahorse in differentiated Th17 cells from HFD mice and the response to adiponectin and compound C were recorded. **(I)** Naïve CD4+ T cells were differentiated into Th17 cells in the presence of adiponectin and frequencies of IL-17+ Th17 cells were measured. Two-tailed non-parametric Mann–Whitney *U*-test was performed for statistical analysis.

In line with the inhibition of pro-inflammatory cytokine production by adiponectin, plasma adiponectin levels were reduced in HFD mice compared to NCD controls (Figure 5A). To elucidate how adiponectin orchestrates its effect on CD4+ T cells, we analyzed the expression of AMP-activated protein kinase (AMPK), the critical nutrient sensor and one of the major abettors of adiponectin action (37) and the expression of its downstream target mTOR. CD4+ T cells from HFD fed mice showed reduced AMPK expression (Figure 5B) and augmented mTOR-pS6 expression (Figure 5C) compared to NCD mice following anti-CD3/anti-CD28 stimulation. This defective AMPK-mTOR axis in combination with the ubiquitous availability of glucose in HFD mice, suggested a dysregulated T cell glycolysis. Such a dysregulated glycolysis was confirmed by measuring the expression of key glycolytic enzymes on purified Th17 cells in NCD and HFD mice with increased expressions of *hk1* (Figure 5D) and *ldh* (Figure 5E) and *pkm* (Figure 5F) on Th17 cells, although the expression of the glucose transporter (*glut1*) and adiponectin receptor was not changed (Supplementary Figures 6A,B) in HFD mice compared to controls.

In order to investigate whether this modulation by adiponectin on Th17 glycolysis is AMPK dependent, the extracellular acidification rate (ECAR) was monitored using the seahorse assay in the presence of compound C, an AMPK inhibitor. Adiponectin significantly reduced the Th17 glycolytic rate and the addition of compound C increased the glycolytic rate of Th17 cells, confirming an AMPK dependency (Figures 5G,H). However, adiponectin had no direct effect on the differentiation of naïve T cells into Th17 cells (Figure 5I).

### Adiponectin Downregulates Th1 Cell Differentiation and Glycolysis Independent of AMPK

**Figure 6:**
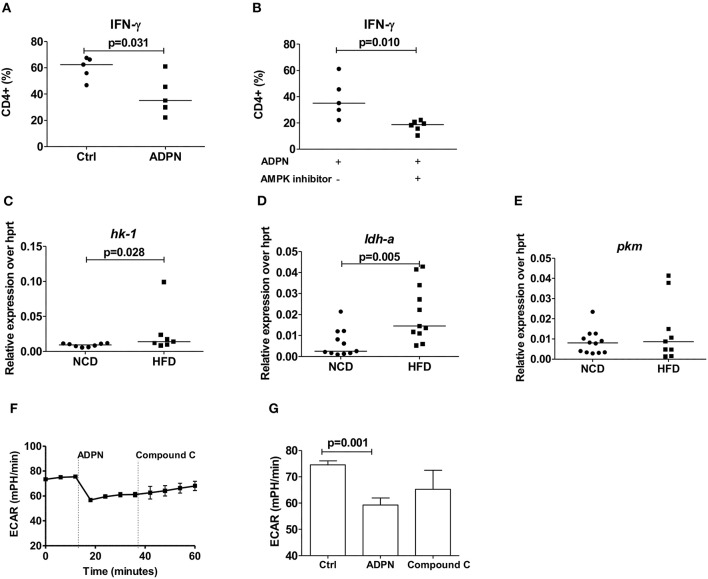
Obesity increases Th1 cell glycolysis and adiponectin dampens Th1 cell glycolysis in an AMPK independent manner. **(A)** Naïve CD4+ T cells were differentiated into Th1 cells in the presence or absence of adiponectin (ADPN) and frequencies of IFN-γ+ T cells are given. **(B)** Naïve T cells were differentiated into Th1 cells in the presence or absence of adiponectin and the AMPK inhibitor compound C. **(C–E)** Relative messenger RNA expression of glycolytic enzymes *hk1* **(C)**, *ldh-a* **(D)**, and *pkm* **(E)** in purified Th1 cells in normal control diet (NCD) and high fat diet (HFD) mice. **(F,G)** Extracellular acidification rate measured by seahorse in differentiated Th1 cells from HFD mice and in response to adiponectin and compound C. Two-tailed non-parametric Mann–Whitney *U*-test was performed for statistical analysis.

We next set out to determine how adiponectin modulates Th1 cell activation. When purified naïve T cells were differentiated in the presence of adiponectin, decreased frequencies of IFN-γ+ T cells (Figure 6A) were noticed. This reduction of IFN-γ+ T cells by adiponectin was independent of AMPK, as the addition of compound C did not reverse the adiponectin-mediated decrease in Th1 differentiation (Figure 6B). In line with its AMPK independent effects, the expression of its major receptor *adipor1* on Th1 cells was comparable between NCD and HFD fed mice (Supplementary Figure 6D). The expression of the glycolytic enzymes *hk-1* (Figure 6C) and *ldh-a* (Figure 6D), but not *pkm* (Figure 6E) and glucose transporter *glut1* (Supplementary Figure 6C) on Th1 cells were increased in the HFD mice compared to NCD mice. However, while adiponectin reduced the glycolytic rate in Th1 cells, this effect was unchanged in the presence of compound C, suggesting a suppression independent of AMPK (Figures 6F,G).

### Filarial Extract Treatment Reduces IFN-γ+ and IL-17+ CD4+ T Cell Frequencies and Increases Adiponectin Release

**Figure 7:**
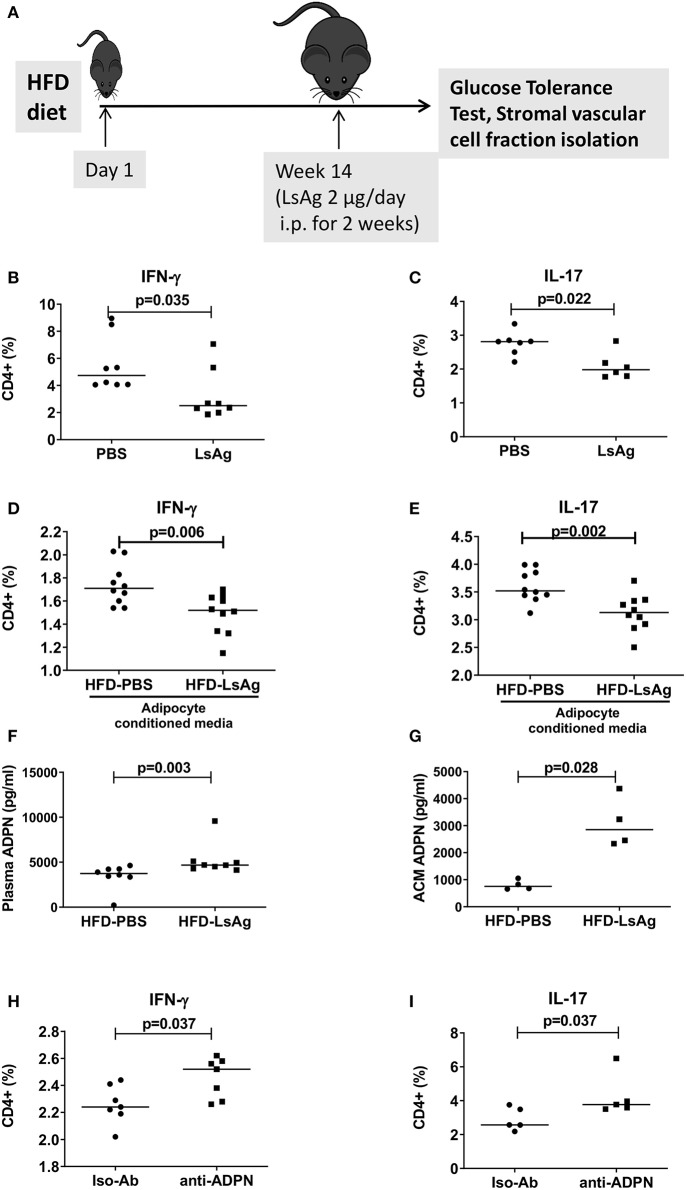
LsAg treatment improves CD4+ T cell inflammation in an adiponectin dependent manner. **(A)** Schematic diagram of LsAg treatment in obese mice. **(B,C)** Frequencies of IFN-γ + **(B)** and IL-17+ **(C)** CD4+ T cells in the adipose tissue of PBS and LsAg treated mice (pooled data from *n* = 2 experiments, 3–4 mice each). **(D,E)** CD4+ T cells positive for IFN-γ **(D)** and IL-17 **(E)** from high fat diet (HFD) mice cultured in the presence of adipocyte conditioned media from PBS and LsAg treated mice. **(F,G)** Plasma and adipocyte conditioned media adiponectin levels in PBS and LsAg-administered HFD mice. **(H,I)** Frequencies of CD4+ T cells from HFD mice after co-culture with anti-adiponectin (anti-ADPN) antibody and isotype control treated adipocyte conditioned media from LsAg treated mice (pooled data from *n* = 3 experiments, 2–3 conditions each). Two-tailed non-parametric Mann–Whitney *U*-test was performed for statistical analysis.

Previous studies demonstrated that helminth infection and helminth-derived products provide a beneficial impact on insulin sensitivity and glucose intolerance (15). In this regard, we demonstrated that filarial adult worm extract (LsAg) improves glucose tolerance and ameliorates adipose tissue inflammation by increasing eosinophils and M2 macrophages within the adipose tissue (19). These results prompted us to investigate if LsAg treatment has an effect on CD4+ T cell inflammation in adipose tissue (Figure 7A). Repeated LsAg administration not only improved glucose intolerance in HFD mice (Supplementary Figures 7A,B), but also significantly reduced IFN-γ+ (Figure 7B) and IL-17+ (Figure 7C) CD4+ T cell frequencies in adipose tissue compared to PBS treated HFD mice even though the body weight was comparable between the groups (data not shown). Further, *in vitro* stimulation of CD4+ T cells with PMA/ionomycin in the presence of adipocyte conditioned media from LsAg treated mice reduced frequencies of IFN-γ+ and IL-17+ T cells compared to CD4+ T cells treated with conditioned media from PBS treated HFD mice, indicating the involvement of a soluble factor (Figures 7D,E). In agreement, LsAg-administered HFD mice had higher adiponectin levels in plasma (Figure 7F) and in adipocyte conditioned media (Figure 7G) compared to PBS treated mice. When adipocyte conditioned media from LsAg-administered HFD mice was treated with adiponectin neutralizing antibodies, the frequencies of IFN-γ and IL-17-producing CD4+ T cells was increased compared to cells that were treated with isotype-treated adiponectin (Figures 7H,I). This suggests that the anti-inflammatory effect of LsAg on IFN-γ and IL-17 cytokine production from CD4+ T cell may be mediated by adiponectin.

## Discussion

Although the positive relationship between adipose tissue inflammation and insulin resistance has been documented earlier (38, 39), literature on the role of adaptive immune cells like T cells of the adipose tissue in the pathogenesis of insulin resistance is still ambiguous. Our study underscores an active involvement of CD4+ and CD8+ Th1 and Th17 cells in obesity, which along with coordinated participation from macrophages and B cells propagates a robust pro-inflammatory milieu. Depleting macrophages and B cells at obesity onset was sufficient to mitigate CD8+ T cell inflammation, while CD4+ T cell inflammation was rather subject to regulation by the adipocyte secreted insulin sensitizer adiponectin. Our study further unveils a novel role of adiponectin in modulating CD4+ T cell mediated cytokine production by restraining T cell glycolysis. While the effects of adiponectin on Th17 glycolysis was orchestrated through AMPK, Th1 differentiation and changes in glycolysis were independent of AMPK.

Obesity increased the activation of CD4 and CD8+ T cells and this activation status was positively correlated with body weight, adipose tissue weight, fasting plasma glucose, and HOMA-IR. In addition, IFN-γ and IL-17 cytokine positive T cells were increasingly observed in adipose tissue from HFD fed mice, which positively correlated with fasting glucose. These results are in agreement with the study of Winer et al., showing increased IFN-γ+ CD4+ T cells in adipose tissue and IL-17+ CD4+ T cells in spleen during obesity (8). However, in contrast to Winer and colleagues (40), we also found increases in IFN-γ+ CD4+ T cells in spleen and IL-17+ CD4+ T cells in adipose tissue during obesity, which may be due to the prolonged HFD treatment in our study, diet composition, and gut microbiota.

Similar to the role of Th1 and Th17 cells during obesity, reports on the role of Th2 cells in obesity are scarce and partly contradictory. McLaughlin et al. reported a negative correlation between insulin resistance and Th2 cells in the blood and adipose tissue (41). However, Weerd et al. observed increased peripheral Th2 cell numbers in morbidly obese subjects compared to normal subjects (42). A third study by Winer et al. showed that CD4+GATA3+ T cells are reduced in the adipose tissue of HFD mice compared to NCD mice, but no differences occur within the spleen (8). Thus, the latter study is in line with our findings demonstrating that no differences in Th2 cell frequencies in the spleens of HFD and NCD groups. Nevertheless, we observed an increase in adipose tissue IL-4+ CD4+ cells in the HFD group and no differences in IL-5+ and IL-13+ CD4+ T cells in the adipose tissue of HFD and NCD mice. Thus, additional studies are required to further elucidate the role of the different Th2 cell subsets during obesity and insulin resistance.

Compared to CD4+ T cells, the role of CD8+ T cells during obesity is less explored despite the fact that CD8+ T cells produce more IFN-γ than CD4+ T cells. Using CD8+ T cell deficient mice and CD8 antibody depletion, Nishimura et al. showed reduced adipose tissue inflammation and insulin resistance (6), while another report showed that transfer of CD8+ T cells into T cell deficient RAG1-HFD mice did not worsen the obesity-associated complications (8). Several studies have shown increased levels of macrophages (43) and B cells (44) in adipose tissue of HFD mice and the critical role of the antigen presenting molecules expressed on their surface in mediating insulin resistance (44, 45). Accordingly, we found increased expression of antigen presenting molecules on macrophages and B cells during obesity and depletion of the antigen presenting cells before the onset of obesity reduced CD8+ T cell inflammation. This could be due to the absence of B cells and macrophages during the initiation of HFD, which in turn could curb CD8+ T cell pro-inflammatory cytokine production, as the infiltration of CD8+ T cells starts a few weeks after the HFD (6).

Obesity was also shown to trigger the expression of NK cell ligand-NCR1 on adipocytes and to lead to higher frequencies of NK cells and increased IFN-γ production by those NK cells within the adipose tissue (46). Similarly, in our study we observed higher frequencies of IFN-γ+ and IL-17+ NK cells in the adipose tissue, while no significant changes in the frequencies of IFN-γ+ and IL-17+ NK cells within the spleen occurred. This suggests that HFD specifically increases frequencies of IFN-γ+ NK cells in the adipose tissue, but their impact on diet-induced insulin resistance and the specific role of IL-17+ NK cells in obesity has yet to be elucidated.

Our results further show that adipocytes from obese mice skew the CD4+ T cells of NCD mice toward a pro-inflammatory phenotype and adipocytes from lean mice reduce the pro-inflammatory cytokine production from CD4+ T cells of obese mice. The fact that CD8+ T cell cytokine production was not affected by either lean or obese adipocytes, support our earlier observation that immune cells of the adipose tissue are primarily responsible for the production of pro-inflammatory cytokines from CD8+ T cells during obesity. Our results concur with those of others who showed that adipocytes express T cell co-stimulatory molecules and genes related to the MHCII pathway (9, 47), whereas MHCI associated genes are not expressed in adipose tissue of obese mice (48). Thus, CD8+ T cells cannot be modulated in a cell-contact dependent manner by adipocytes.

In the case of CD4+ T cells, adipocytes modulated cytokine production in a cell-contact independent mechanism. We found that although palmitic and oleic acid constitute the major adipose tissue lipids, *in vitro* treatment of NCD mice-derived CD4+ T cells with palmitic acid moderately increased Th1 cells whereas *in vitro* treatment of obese CD4+ T cells with oleic acid slightly but significantly reduced Th1 cells. With regard to adiponectin and leptin, two critical mediators of metabolic homeostasis in adipose tissue and possible regulators of CD4+ T cell function, leptin treatment increased IFN-γ+ CD4+ T cell frequencies in NCD mice and slightly reduced Th1 and Th17 frequencies during obesity. Previous reports showed that adoptive transfer of autoreactive T cells in leptin deficient mice reduced pathogenicity and decreased frequencies of Th1 and Th17 cells in the experimental autoimmune encephalomyelitis model (49). On the other hand, our study demonstrates an inhibitory effect of adiponectin on IFN-γ and IL-17 production from CD4+ T cells. To our knowledge there is no consensus in the literature on whether adiponectin antagonizes or propagates T cell inflammation in other diseases. While adiponectin treatment has been shown to reduce T cell specific CXCR3 expression and the recruitment of T cells to the atherogenic sites (50), Cheng et al. showed that stimulation of human CD4+ T cells with anti-CD3/anti-CD28 in the presence of adiponectin increases mRNA expression and protein secretion of IFN-γ on day 3 without affecting T cell proliferation in a concentration dependent manner (22). Further, the same study has documented that adiponectin treatment increases IFN-γ secretion in a p38 dependent and AMPK independent manner from human CD4+ T cells (22). These results argue in favor of a highly context specific and selective modulation of the immune cells by adiponectin. To the best of our knowledge, the present study is the first report showing an adiponectin mediated modulation of CD4+ T cells during obesity.

Despite the fact that immune cells are major consumers of nutrients and energy, the mechanisms of how metabolic reprogramming can shape the function of immune cells are only beginning to emerge (12, 51). Hence we investigated, if nutritional overload reshapes the functions of obesity-derived T cells and the function of adiponectin in this process. Adiponectin decreased the differentiation of purified naïve CD4+ T cells into Th1 cells in an AMPK independent manner, while there was no effect of adiponectin on the differentiation of Th17 cells. Adiponectin also significantly suppressed glycolysis in both Th1 and Th17 cells, the former being executed in an AMPK independent and the latter via an AMPK dependent manner. Although the effects of adiponectin as a potent insulin sensitizer and immune modulator are well-known, we show for the first time that adiponectin has the ability to modulate T cell functions in the context of obesity. Saucillo et al. have earlier demonstrated leptin as the essential link between T cell metabolism and nutritional status. Addition of leptin to T cell cultures of fasted animals or leptin injections to fasted animals rescued metabolic and functional defects of effector T cells (13). Further, the same group has demonstrated a selective and cell intrinsic requirement for leptin during autoimmunity to upregulate glucose metabolism so that the effector functions of T cells are controlled (14).

While diet-induced inflammation is a known etiological factor for insulin resistance, our results suggest that metabolic dysfunction and lower adiponectin levels combined with increased Th1 and Th17 glycolysis lead to an expansion of IFN-γ+ and IL-17+ T cells that contribute to obesity. However, detailed kinetic studies are required to decipher whether these metabolic changes are preceding the inflammatory responses during the onset of HFD or whether they occur in parallel.

Another important finding of our study is that the beneficial effect of LsAg on glucose intolerance and the amelioration of obesity may be mediated via adiponectin. LsAg treatment reduced IFN-γ and IL-17+ CD4+ T cell frequencies during obesity. In agreement with our results, a recent report by Su et al. showed that another helminth, *Heligmosomoides polygyrus* increased *gata-3* and reduced *T-bet* and *rorc* gene expression, indicating an expansion of Th2 cells and reduction of Th1 and Th17 cells, increased secretion of IL-10, IL-4 and decreased IFN-γ and IL-17 from mesenteric lymph node cells of helminth-infected, obese mice compared to controls (52). Such an induction of type 2 immune responses was also observed by LsAg treatment in obese mice, which expanded FoxP3+ regulatory T cells, M2 macrophages, ILC2s and eosinophils and reduced classically activated M1 macrophages in adipose tissue without altering adipocyte size in obese mice (19). Our current study further showed an increase of adiponectin both at circulating levels and also in adipocyte supernatant from LsAg-treated mice and that adipose tissue of LsAg-treated mice reduced IFN-γ+ and IL-17+ T cell frequencies in an adiponectin-dependent manner. Thus, these results are in accordance with the SUGARSPIN trial findings where anti-helminthic therapy was shown to increase the systemic leptin/adiponectin ratio in helminth-infected subjects (53), indicating that this pathway may be also of importance during human helminth infection. As several other studies have also shown that helminth infection or helminth-derived products improve adipose tissue inflammation by inducing a type 2 immune response (20), it can be speculated that helminths and their products dampen in general the pro-inflammatory cytokine production by CD4+ T cells by increasing adiponectin production from the adipocytes. This will be now analyzed in a human study including obese and lean individuals that are infected with the filarial nematodes *Onchocerca volvulus* or *Mansonella perstans*.

In summary, our study shows that obesity-induced increase of IFN-γ and IL-17 positive T cells is differentially regulated. This study identified a novel role for adiponectin in restraining the Th1 and Th17 cell glycolysis to mitigate inflammation and also unravels an adiponectin mediated mechanism that helminths and helminth-derived products may use to reduce adipose tissue T cell inflammation (see Graphical Abstract).

## Data Availability Statement

All datasets generated for this study are included in the article/Supplementary Material.

## Ethics Statement

The animal study was reviewed and approved by all protocols were approved by the Landesamt für Natur, Umwelt und Verbraucherschutz, Cologne, Germany (84-02.04.2016.A331).

## Author Contributions

JS and MH designed the study. JS, SF, IK, WS, A-LN, and VS performed the experiments. JS and MH analyzed the data and interpreted the results. CW and AH contributed to the design of the experiments and reviewed and edited the manuscript. JS, IK, and MH wrote the manuscript.

### Conflict of Interest

The authors declare that the research was conducted in the absence of any commercial or financial relationships that could be construed as a potential conflict of interest.

## Supplementary Material

Supplementary Figure 1 Obesity triggers glucose and insulin intolerance. **(A)** Flow chart of the experimental study design. Body weight **(B)**, adipose tissue weight **(C)**, glucose levels during the glucose tolerance test **(D)** and insulin tolerance test **(F)** as well as the calculated area under the curve (AUC) from the glucose **(E)** and insulin tolerance test **(G)** of control and HFD mice. Two-tailed non-parametric Mann–Whitney *U*-test was performed for statistical analysis. Single experiment with *n* = 8 per group. ^**^*p* < 0.01.

Supplementary Figure 2 Gating strategy for CD4+ and CD8+ T cells. IFN-γ and IL-17 cytokine producing CD4+ and CD8+ T cells within the adipose tissue were identified after *in vitro* PMA/Ionomycin stimulation by flow cytometry. The complete cell population was selected and doublet cells were excluded by FSC-W characteristics. Based on FSC-A and SSC, lymphocytes were selected and T cells were identified based on CD4 and CD8 positivity. Intracellular expression of IFN-γ and IL-17 were gated from CD4+ and CD8+ cells via the fluorescence minus one approach.

Supplementary Figure 3 Obesity partly increases IFN-γ and IL-17 cytokine producing T cells in the spleen. **(A–D)** Frequency of IFN-γ+ **(A,C)** and IL-17+ **(B,D)** CD4+ and CD8+ T cells from spleen (pooled data from *n* = 2 experiments, 4–6 mice each). Two-tailed non-parametric Mann–Whitney *U*-test was performed for statistical analysis.

Supplementary Figure 4 Obesity increases IFN-γ+ and IL-17+ NK cells in the adipose tissue, but has no impact on IL-5+ and IL-13+ Th2 cell frequencies. **(A–D)** Frequency of IFN-γ+ **(A,C)** and IL-17+ **(B,D)** NK cells in the adipose tissue **(A,B)** and spleen **(C,D)**. **(E–J)** Frequencies of IL-4+ **(E,H)** and IL-5+ **(F,I)** and IL-13+ **(G,J)** CD4 T cells in adipose tissue **(E–G)** and spleen **(H–J)**. Pooled data from *n* = 2 experiments with 3–4 mice each. Two-tailed non-parametric Mann–Whitney *U*-test was performed for statistical analysis.

Supplementary Figure 5 Obese adipocytes do not alter CD8+ T cell cytokine production. CD8+ T cells from normal control diet (NCD) and high fat diet (HFD) mice were isolated and cultured with adipocytes from lean and obese mice with anti-CD3/CD28. **(A,B)** Frequencies of IFN-γ **(A)** and IL-17 **(B)** positive NCD CD8+ T cells upon adipocyte co-culture. **(C,D)** IFN-γ **(C)** and IL-17 **(D)** positive HFD CD8+ T cells frequencies upon adipocyte co-culture. Two-tailed non-parametric Mann–Whitney *U*-test was performed for statistical analysis.

Supplementary Figure 6 Obesity does not influence adiponectin receptor or glucose transporter expression on Th1 and Th17 cells. **(A–D)** Relative mRNA expression of glucose transporter (*glut-1*) and adiponectin receptor 1 (*adipor1*) on purified Th17 **(A,B)** and Th1 cells **(C,D)** in normal control diet (NCD) and high fat diet (HFD) mice. Two-tailed non-parametric Mann–Whitney *U*-test was performed for statistical analysis.

Supplementary Figure 7 LsAg improves glucose intolerance in obese mice. **(A)** Blood glucose concentrations over time after i.p. glucose challenge in PBS- and LsAg-administered high fat diet (HFD) mice. **(B)** Calculated area under curve (AUC) from the course of the glucose tolerance test. ^*^*p* < 0.05.

## References

[1] Ng M Fleming T Robinson M Thomson B Graetz N Margono C. Global, regional, and national prevalence of overweight and obesity in children and adults during 1980-2013: a systematic analysis for the Global Burden of Disease Study 2013. Lancet. (2014) 384:766–81. 10.1016/S0140-6736(14)60460-8 24880830 PMC4624264

[2] Lumeng CN. Innate immune activation in obesity. Mol Aspects Med. (2013) 34:12–29. 10.1016/j.mam.2012.10.002 23068074 PMC3888776

[3] Weisberg SP Mccann D Desai M Rosenbaum M Leibel RL Ferrante AW Jr. Obesity is associated with macrophage accumulation in adipose tissue. J Clin Invest. (2003) 112:1796–808. 10.1172/JCI200319246 14679176 PMC296995

[4] Xu H Barnes GT Yang Q Tan G Yang D Chou CJ. Chronic inflammation in fat plays a crucial role in the development of obesity-related insulin resistance. J Clin Invest. (2003) 112:1821–30. 10.1172/JCI200319451 14679177 PMC296998

[5] Kintscher U Hartge M Hess K Foryst-Ludwig A Clemenz M Wabitsch M. T-lymphocyte infiltration in visceral adipose tissue: a primary event in adipose tissue inflammation and the development of obesity-mediated insulin resistance. Arterioscler Thromb Vasc Biol. (2008) 28:1304–10. 10.1161/ATVBAHA.108.165100 18420999

[6] Nishimura S Manabe I Nagasaki M Eto K Yamashita H Ohsugi M. CD8+ effector T cells contribute to macrophage recruitment and adipose tissue inflammation in obesity. Nat Med. (2009) 15:914–20. 10.1038/nm.1964 19633658

[7] Yang H Youm YH Vandanmagsar B Ravussin A Gimble JM Greenway F. Obesity increases the production of proinflammatory mediators from adipose tissue T cells and compromises TCR repertoire diversity: implications for systemic inflammation and insulin resistance. J Immunol. (2010) 185:1836–45. 10.4049/jimmunol.1000021 20581149 PMC4829921

[8] Winer S Chan Y Paltser G Truong D Tsui H Bahrami J. Normalization of obesity-associated insulin resistance through immunotherapy. Nat Med. (2009) 15:921–9. 10.1038/nm.2001 19633657 PMC3063199

[9] Deng T Lyon CJ Minze LJ Lin J Zou J Liu JZ. Class II major histocompatibility complex plays an essential role in obesity-induced adipose inflammation. Cell Metab. (2013) 17:411–22. 10.1016/j.cmet.2013.02.009 23473035 PMC3619392

[10] Procaccini C De Rosa V Galgani M Carbone F La Rocca C Formisano L. Role of adipokines signaling in the modulation of T cells function. Front Immunol. (2013) 4:332. 10.3389/fimmu.2013.00332 24151494 PMC3799205

[11] De Jong AJ Kloppenburg M Toes RE Ioan-Facsinay A. Fatty acids, lipid mediators, and T-cell function. Front Immunol. (2014) 5:483. 10.3389/fimmu.2014.00483 25352844 PMC4195378

[12] Buck MD Sowell RT Kaech SM Pearce EL. Metabolic Instruction of Immunity. Cell. (2017) 169:570–86. 10.1016/j.cell.2017.04.004 28475890 PMC5648021

[13] Saucillo DC Gerriets VA Sheng J Rathmell JC Maciver NJ. Leptin metabolically licenses T cells for activation to link nutrition and immunity. J Immunol. (2014) 192:136–44. 10.4049/jimmunol.1301158 24273001 PMC3872216

[14] Gerriets VA Danzaki K Kishton RJ Eisner W Nichols AG Saucillo DC. Leptin directly promotes T-cell glycolytic metabolism to drive effector T-cell differentiation in a mouse model of autoimmunity. Eur J Immunol. (2016) 46:1970–83. 10.1002/eji.201545861 27222115 PMC5154618

[15] Surendar J Indulekha K Hoerauf A Hübner MP. Immunomodulation by helminths: Similar impact on type 1 and type 2 diabetes? Parasite Immunol. (2017) 39:1–15. 10.1111/pim.12401 27862000

[16] Aravindhan V Mohan V Surendar J Muralidhara Rao M Pavankumar N Deepa M. Decreased prevalence of lymphatic filariasis among diabetic subjects associated with a diminished pro-inflammatory cytokine response (CURES 83). PLoS Negl Trop Dis. (2010) 4:e707. 10.1371/journal.pntd.0000707 20559443 PMC2886036

[17] Chen Y Lu J Huang Y Wang T Xu Y Xu M. Association of previous schistosome infection with diabetes and metabolic syndrome: a cross-sectional study in rural China. J Clin Endocrinol Metab. (2013) 98:E283–7. 10.1210/jc.2012-2517 23275524

[18] Hussaarts L Garcia-Tardon N Van Beek L Heemskerk MM Haeberlein S Van Der Zon GC. Chronic helminth infection and helminth-derived egg antigens promote adipose tissue M2 macrophages and improve insulin sensitivity in obese mice. FASEB J. (2015) 29:3027–39. 10.1096/fj.14-266239 25852044

[19] Berbudi A Surendar J Ajendra J Gondorf F Schmidt D Neumann AL. Filarial infection or antigen administration improves glucose tolerance in diet-induced obese mice. J Innate Immun. (2016) 8:601–16. 10.1159/000448401 27544668 PMC6743339

[20] Berbudi A Ajendra J Wardani AP Hoerauf A Hübner MP. Parasitic helminths and their beneficial impact on type 1 and type 2 diabetes. Diabetes Metab Res Rev. (2016) 32:238–50. 10.1002/dmrr.2673 26119261

[21] Hübner MP Stocker JT Mitre E. Inhibition of type 1 diabetes in filaria-infected non-obese diabetic mice is associated with a T helper type 2 shift and induction of FoxP3+ regulatory T cells. Immunology. (2009) 127:512–22. 10.1111/j.1365-2567.2008.02958.x 19016910 PMC2729528

[22] Cheng X Folco EJ Shimizu K Libby P. Adiponectin induces pro-inflammatory programs in human macrophages and CD4+ T cells. J Biol Chem. (2012) 287:36896–904. 10.1074/jbc.M112.409516 22948153 PMC3481292

[23] De Rosa V Procaccini C Cali G Pirozzi G Fontana S Zappacosta S. A key role of leptin in the control of regulatory T cell proliferation. Immunity. (2007) 26:241–55. 10.1016/j.immuni.2007.01.011 17307705

[24] Hoo RL Shu L Cheng KK Wu X Liao B Wu D. Adipocyte fatty acid binding protein potentiates toxic lipids-induced endoplasmic reticulum stress in macrophages via inhibition of Janus Kinase 2-dependent autophagy. Sci Rep. (2017) 7:40657. 10.1038/srep40657 28094778 PMC5240568

[25] Angela M Endo Y Asou HK Yamamoto T Tumes DJ Tokuyama H. Fatty acid metabolic reprogramming via mTOR-mediated inductions of PPARgamma directs early activation of T cells. Nat Commun. (2016) 7:13683. 10.1038/ncomms13683 27901044 PMC5141517

[26] Klein-Wieringa IR Kloppenburg M Bastiaansen-Jenniskens YM Yusuf E Kwekkeboom JC El-Bannoudi H. The infrapatellar fat pad of patients with osteoarthritis has an inflammatory phenotype. Ann Rheum Dis. (2011) 70:851–7. 10.1136/ard.2010.140046 21242232

[27] Ioan-Facsinay A Kwekkeboom JC Westhoff S Giera M Rombouts Y Van Harmelen V. Adipocyte-derived lipids modulate CD4+ T-cell function. Eur J Immunol. (2013) 43:1578–87. 10.1002/eji.201243096 23504601

[28] Feng B Jiao P Nie Y Kim T Jun D Van Rooijen N. Clodronate liposomes improve metabolic profile and reduce visceral adipose macrophage content in diet-induced obese mice. PLoS ONE. (2011) 6:e24358. 10.1371/journal.pone.0024358 21931688 PMC3171445

[29] Guo L Kapur R Aslam R Speck ER Zufferey A Zhao Y. CD20+ B-cell depletion therapy suppresses murine CD8+ T-cell-mediated immune thrombocytopenia. Blood. (2016) 127:735–8. 10.1182/blood-2015-06-655126 26556550

[30] Omenetti S Bussi C Metidji A Iseppon A Lee S Tolaini M. The intestine harbors functionally distinct homeostatic tissue-resident and inflammatory Th17 cells. Immunity. (2019) 51:77–89 e76. 10.1016/j.immuni.2019.05.004 31229354 PMC6642154

[31] Saraiva M Christensen JR Veldhoen M Murphy TL Murphy KM O'garra A. Interleukin-10 production by Th1 cells requires interleukin-12-induced STAT4 transcription factor and ERK MAP kinase activation by high antigen dose. Immunity. (2009) 31:209–19. 10.1016/j.immuni.2009.05.012 19646904 PMC2791889

[32] Flaherty S Reynolds JM Mouse naive CD4+ T cell isolation and *in vitro* differentiation into T cell subsets. J Vis Exp. (2015) e52739 10.3791/52739 PMC4541570 25938923

[33] Ajendra J Specht S Neumann AL Gondorf F Schmidt D Gentil K. ST2 Deficiency does not impair Type 2 immune responses during chronic filarial infection but leads to an increased microfilaremia due to an impaired splenic microfilarial clearance. PLoS ONE. (2014) 9:e93072. 10.1371/journal.pone.0093072 24663956 PMC3963995

[34] De Boer AA Monk JM Liddle DM Hutchinson AL Power KA Ma DW. Fish-oil-derived n-3 polyunsaturated fatty acids reduce NLRP3 inflammasome activity and obesity-related inflammatory cross-talk between adipocytes and CD11b(+) macrophages. J Nutr Biochem. (2016) 34:61–72. 10.1016/j.jnutbio.2016.04.004 27208584

[35] Matthews DR Hosker JP Rudenski AS Naylor BA Treacher DF Turner RC. Homeostasis model assessment: insulin resistance and beta-cell function from fasting plasma glucose and insulin concentrations in man. Diabetologia. (1985) 28:412–9. 10.1007/BF00280883 3899825

[36] Lopez-Jaramillo P Gomez-Arbelaez D Lopez-Lopez J Lopez-Lopez C Martinez-Ortega J Gomez-Rodriguez A. The role of leptin/adiponectin ratio in metabolic syndrome and diabetes. Horm Mol Biol Clin Investig. (2014) 18:37–45. 10.1515/hmbci-2013-0053 25389999

[37] Hardie DG Ross FA Hawley SA. AMPK: a nutrient and energy sensor that maintains energy homeostasis. Nat Rev Mol Cell Biol. (2012) 13:251–62. 10.1038/nrm3311 22436748 PMC5726489

[38] Shoelson SE Lee J Goldfine AB. Inflammation and insulin resistance. J Clin Invest. (2006) 116:1793–801. 10.1172/JCI29069 16823477 PMC1483173

[39] De Luca C Olefsky JM. Inflammation and insulin resistance. FEBS Lett. (2008) 582:97–105. 10.1016/j.febslet.2007.11.057 18053812 PMC2246086

[40] Winer S Paltser G Chan Y Tsui H Engleman E Winer D. Obesity predisposes to Th17 bias. Eur J Immunol. (2009) 39:2629–35. 10.1002/eji.200838893 19662632

[41] Mclaughlin T Liu LF Lamendola C Shen L Morton J Rivas H. T-cell profile in adipose tissue is associated with insulin resistance and systemic inflammation in humans. Arterioscler Thromb Vasc Biol. (2014) 34:2637–43. 10.1161/ATVBAHA.114.304636 25341798 PMC4445971

[42] Van Der Weerd K Dik WA Schrijver B Schweitzer DH Langerak AW Drexhage HA. Morbidly obese human subjects have increased peripheral blood CD4+ T cells with skewing toward a Treg- and Th2-dominated phenotype. Diabetes. (2012) 61:401–8. 10.2337/db11-1065 22228716 PMC3266399

[43] Olefsky JM Glass CK. Macrophages, inflammation, and insulin resistance. Annu Rev Physiol. (2010) 72:219–46. 10.1146/annurev-physiol-021909-135846 20148674

[44] Winer DA Winer S Shen L Wadia PP Yantha J Paltser G. B cells promote insulin resistance through modulation of T cells and production of pathogenic IgG antibodies. Nat Med. (2011) 17:610–7. 10.1038/nm.2353 21499269 PMC3270885

[45] Cho KW Morris DL Delproposto JL Geletka L Zamarron B Martinez-Santibanez G. An MHC II-dependent activation loop between adipose tissue macrophages and CD4+ T cells controls obesity-induced inflammation. Cell Rep. (2014) 9:605–17. 10.1016/j.celrep.2014.09.004 25310975 PMC4252867

[46] Wensveen FM Jelencic V Valentic S Sestan M Wensveen TT Theurich S. NK cells link obesity-induced adipose stress to inflammation and insulin resistance. Nat Immunol. (2015) 16:376–85. 10.1038/ni.3120 25729921

[47] Meijer K De Vries M Al-Lahham S Bruinenberg M Weening D Dijkstra M. Human primary adipocytes exhibit immune cell function: adipocytes prime inflammation independent of macrophages. PLoS ONE. (2011) 6:e17154. 10.1371/journal.pone.0017154 21448265 PMC3063154

[48] Xiao L Yang X Lin Y Li S Jiang J Qian S. Large adipocytes function as antigen-presenting cells to activate CD4(+) T cells via upregulating MHCII in obesity. Int J Obes (Lond). (2016) 40:112–20. 10.1038/ijo.2015.145 26248660 PMC4722243

[49] Galgani M Procaccini C De Rosa V Carbone F Chieffi P La Cava A. Leptin modulates the survival of autoreactive CD4+ T cells through the nutrient/energy-sensing mammalian target of rapamycin signaling pathway. J Immunol. (2010) 185:7474–9. 10.4049/jimmunol.1001674 21078910

[50] Okamoto Y Folco EJ Minami M Wara AK Feinberg MW Sukhova GK. Adiponectin inhibits the production of CXC receptor 3 chemokine ligands in macrophages and reduces T-lymphocyte recruitment in atherogenesis. Circ Res. (2008) 102:218–25. 10.1161/CIRCRESAHA.107.164988 17991878

[51] O'neill LA Kishton RJ Rathmell J. A guide to immunometabolism for immunologists. Nat Rev Immunol. (2016) 16:553–65. 10.1038/nri.2016.70 27396447 PMC5001910

[52] Su CW Chen CY Li Y Long SR Massey W Kumar DV. Helminth infection protects against high fat diet-induced obesity via induction of alternatively activated macrophages. Sci Rep. (2018) 8:4607. 10.1038/s41598-018-22920-7 29545532 PMC5854586

[53] Tahapary DL De Ruiter K Martin I Brienen EAT Van Lieshout L Djuardi Y. Effect of anthelmintic treatment on leptin, adiponectin and leptin to adiponectin ratio: a randomized-controlled trial. Nutr Diabetes. (2017) 7:e289. 10.1038/nutd.2017.37 29035384 PMC5678209

