# Supplementary figures and images for: Adiponectin Limits IFN-γ and IL-17 Producing CD4 T Cells in Obesity by Restraining Cell Intrinsic Glycolysis
Source: Front Immunol. 2019 Oct 29;10:2555. doi: 10.3389/fimmu.2019.02555 (PMC6828851; doi:10.3389/fimmu.2019.02555)

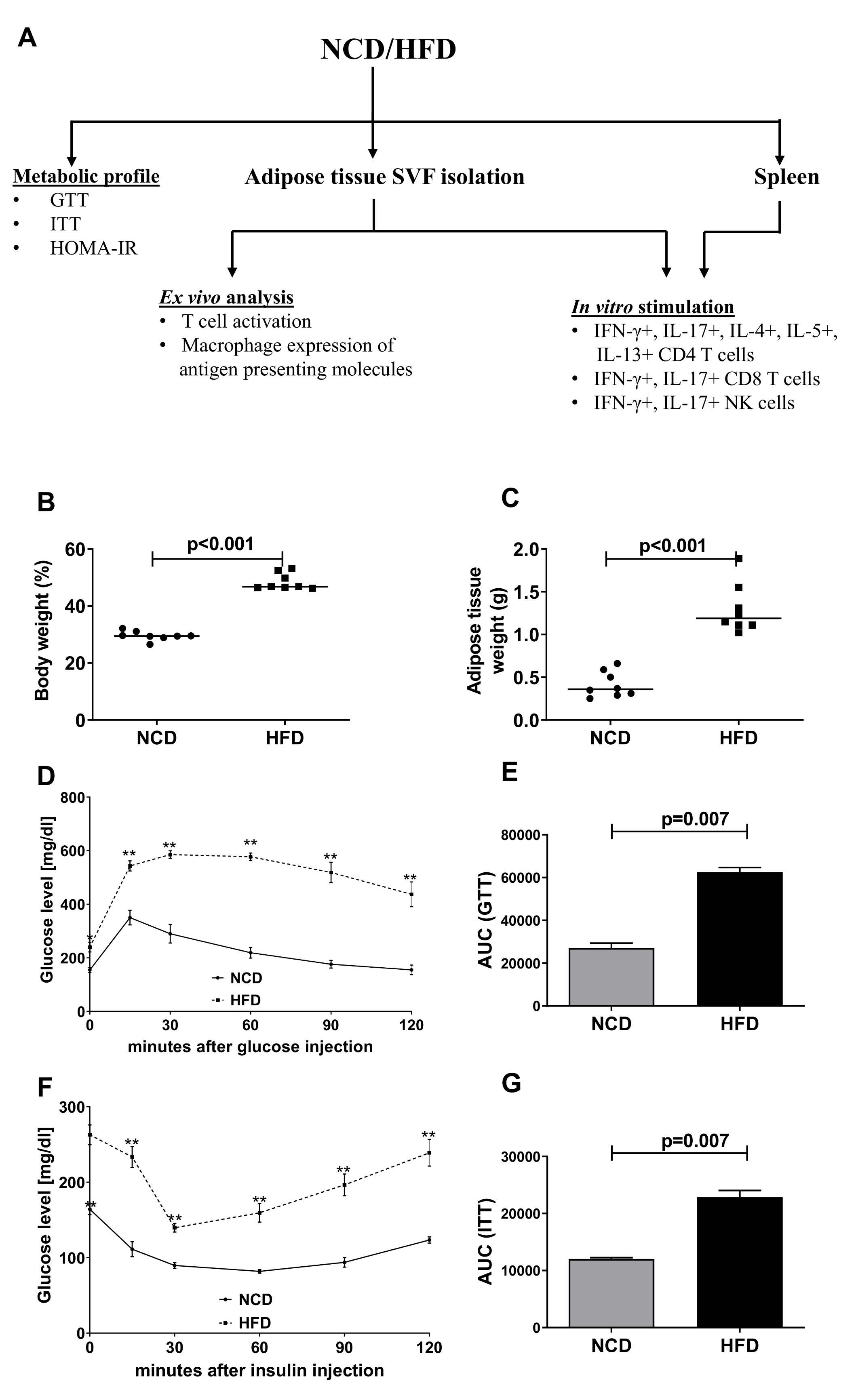

Supplement: Supplementary Figure 1 — Obesity triggers glucose and insulin intolerance. (A) Flow chart of the experimental study design. Body weight (B), adipose tissue weight (C), glucose levels during the glucose tolerance test (D) and insulin tolerance test (F) as well as the calculated area under the curve (AUC) from the glucose (E) and insulin tolerance test (G) of control and HFD mice. Two-tailed non-parametric Mann–Whitney U-test was performed for statistical analysis. Single experiment with n = 8 per group. **p < 0.01. [file Image_1.TIFF]

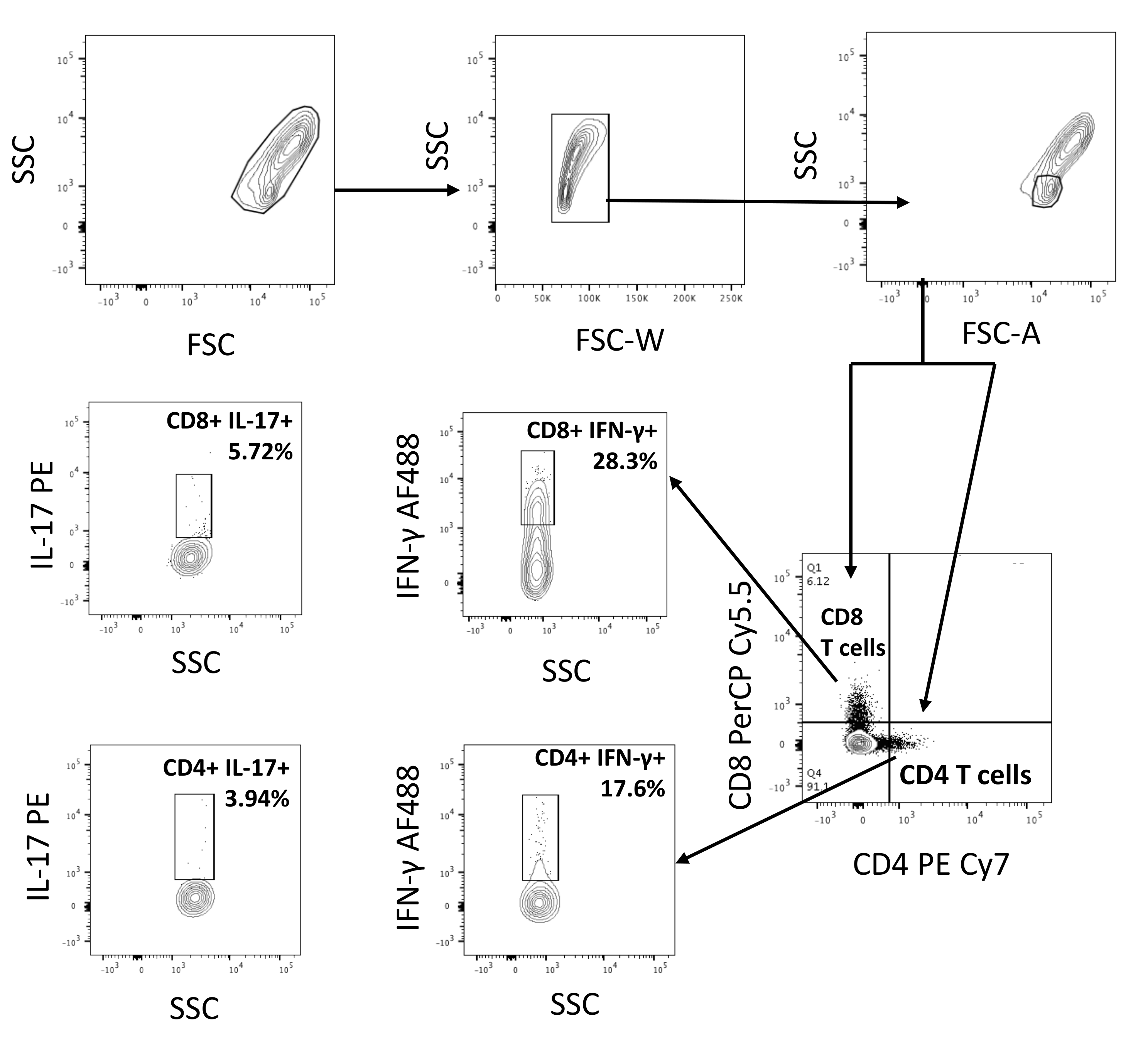

Supplement: Supplementary Figure 2 — Gating strategy for CD4+ and CD8+ T cells. IFN-γ and IL-17 cytokine producing CD4+ and CD8+ T cells within the adipose tissue were identified after in vitro PMA/Ionomycin stimulation by flow cytometry. The complete cell population was selected and doublet cells were excluded by FSC-W characteristics. Based on FSC-A and SSC, lymphocytes were selected and T cells were identified based on CD4 and CD8 positivity. Intracellular expression of IFN-γ and IL-17 were gated from CD4+ and CD8+ cells via the fluorescence minus one approach. [file Image_2.TIFF]

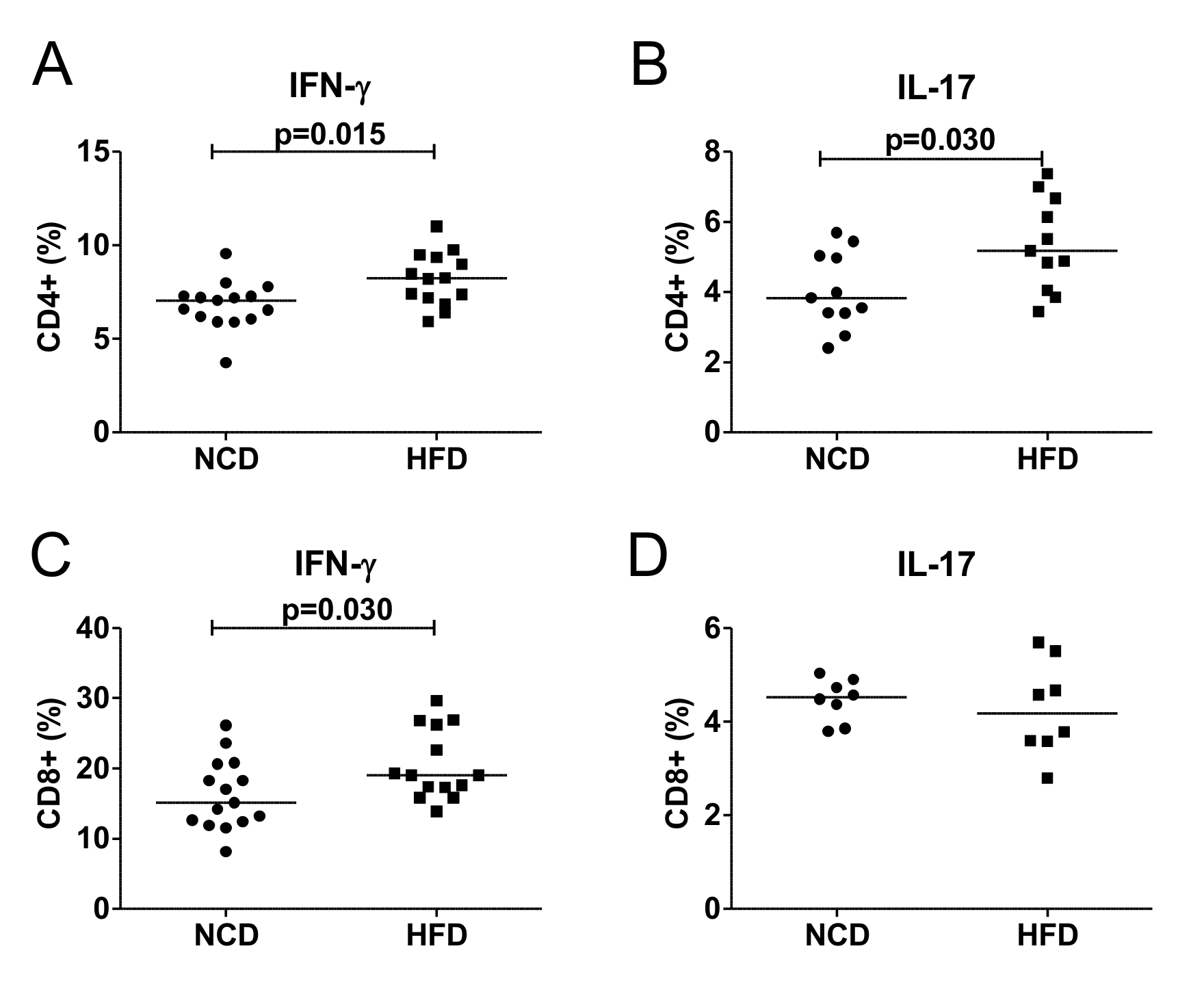

Supplement: Supplementary Figure 3 — Obesity partly increases IFN-γ and IL-17 cytokine producing T cells in the spleen. (A–D) Frequency of IFN-γ+ (A,C) and IL-17+ (B,D) CD4+ and CD8+ T cells from spleen (pooled data from n = 2 experiments, 4–6 mice each). Two-tailed non-parametric Mann–Whitney U-test was performed for statistical analysis. [file Image_3.TIFF]

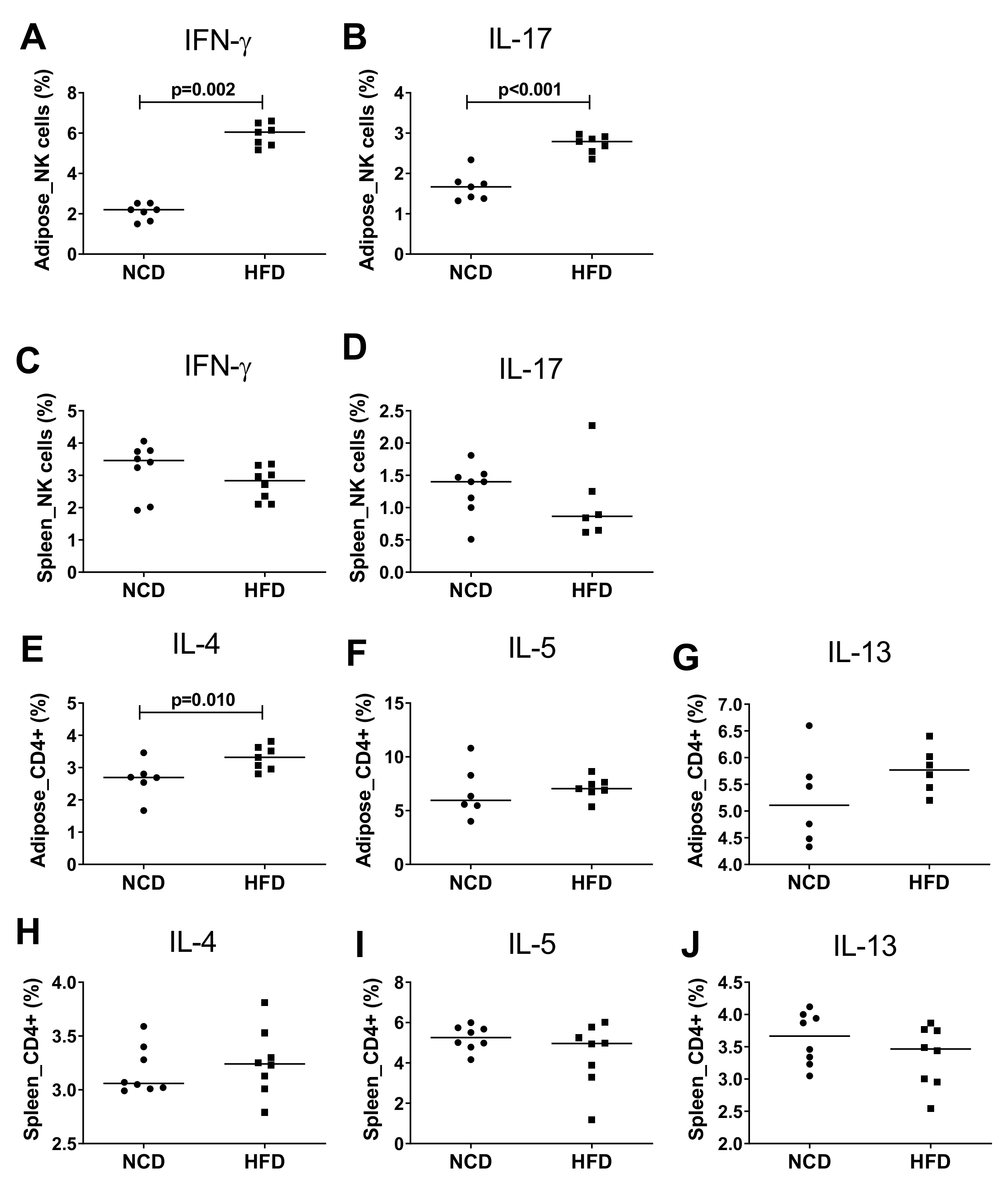

Supplement: Supplementary Figure 4 — Obesity increases IFN-γ+ and IL-17+ NK cells in the adipose tissue, but has no impact on IL-5+ and IL-13+ Th2 cell frequencies. (A–D) Frequency of IFN-γ+ (A,C) and IL-17+ (B,D) NK cells in the adipose tissue (A,B) and spleen (C,D). (E–J) Frequencies of IL-4+ (E,H) and IL-5+ (F,I) and IL-13+ (G,J) CD4 T cells in adipose tissue (E–G) and spleen (H–J). Pooled data from n = 2 experiments with 3–4 mice each. Two-tailed non-parametric Mann–Whitney U-test was performed for statistical analysis. [file Image_4.tiff]

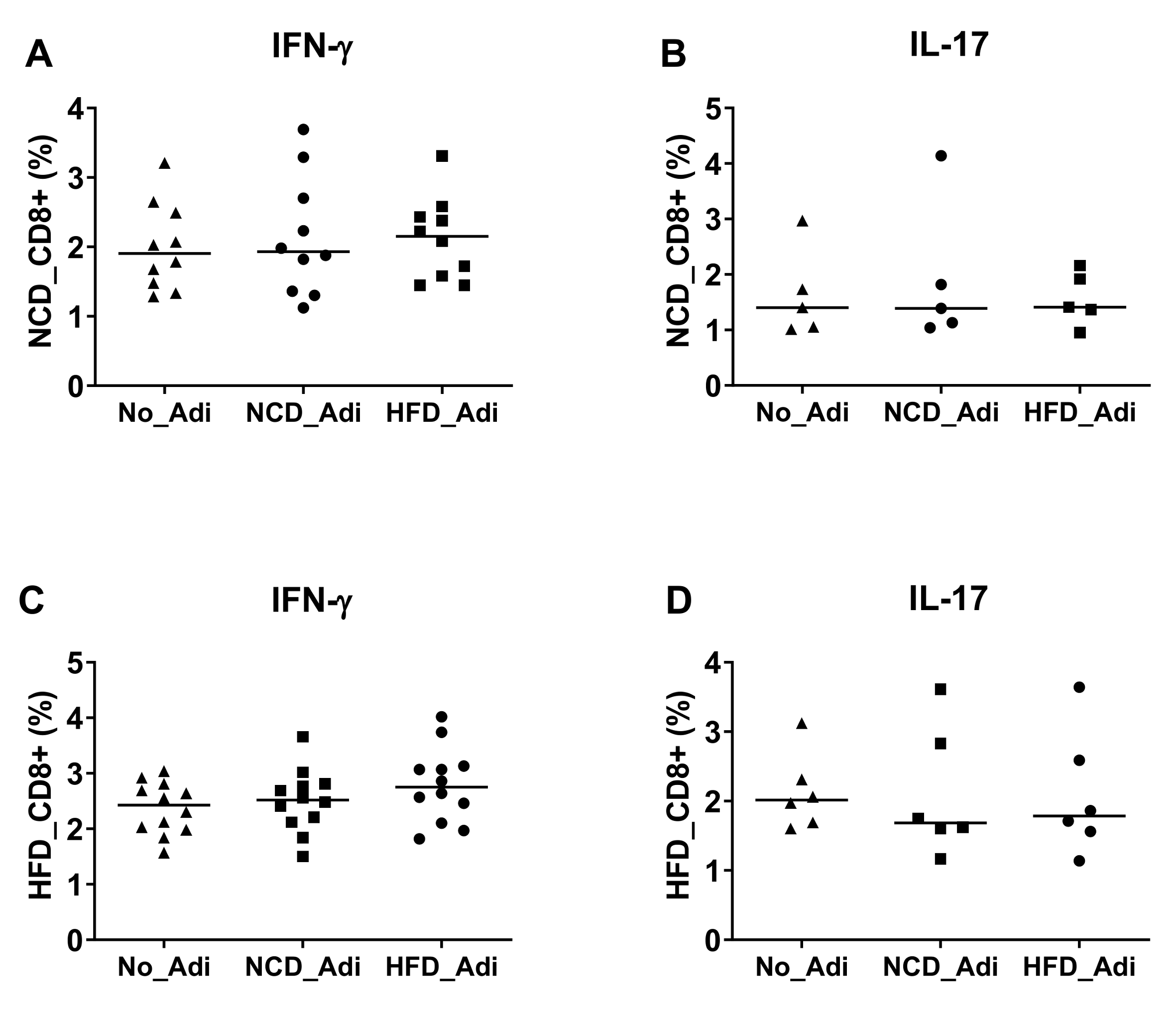

Supplement: Supplementary Figure 5 — Obese adipocytes do not alter CD8+ T cell cytokine production. CD8+ T cells from normal control diet (NCD) and high fat diet (HFD) mice were isolated and cultured with adipocytes from lean and obese mice with anti-CD3/CD28. (A,B) Frequencies of IFN-γ (A) and IL-17 (B) positive NCD CD8+ T cells upon adipocyte co-culture. (C,D) IFN-γ (C) and IL-17 (D) positive HFD CD8+ T cells frequencies upon adipocyte co-culture. Two-tailed non-parametric Mann–Whitney U-test was performed for statistical analysis. [file Image_5.tiff]

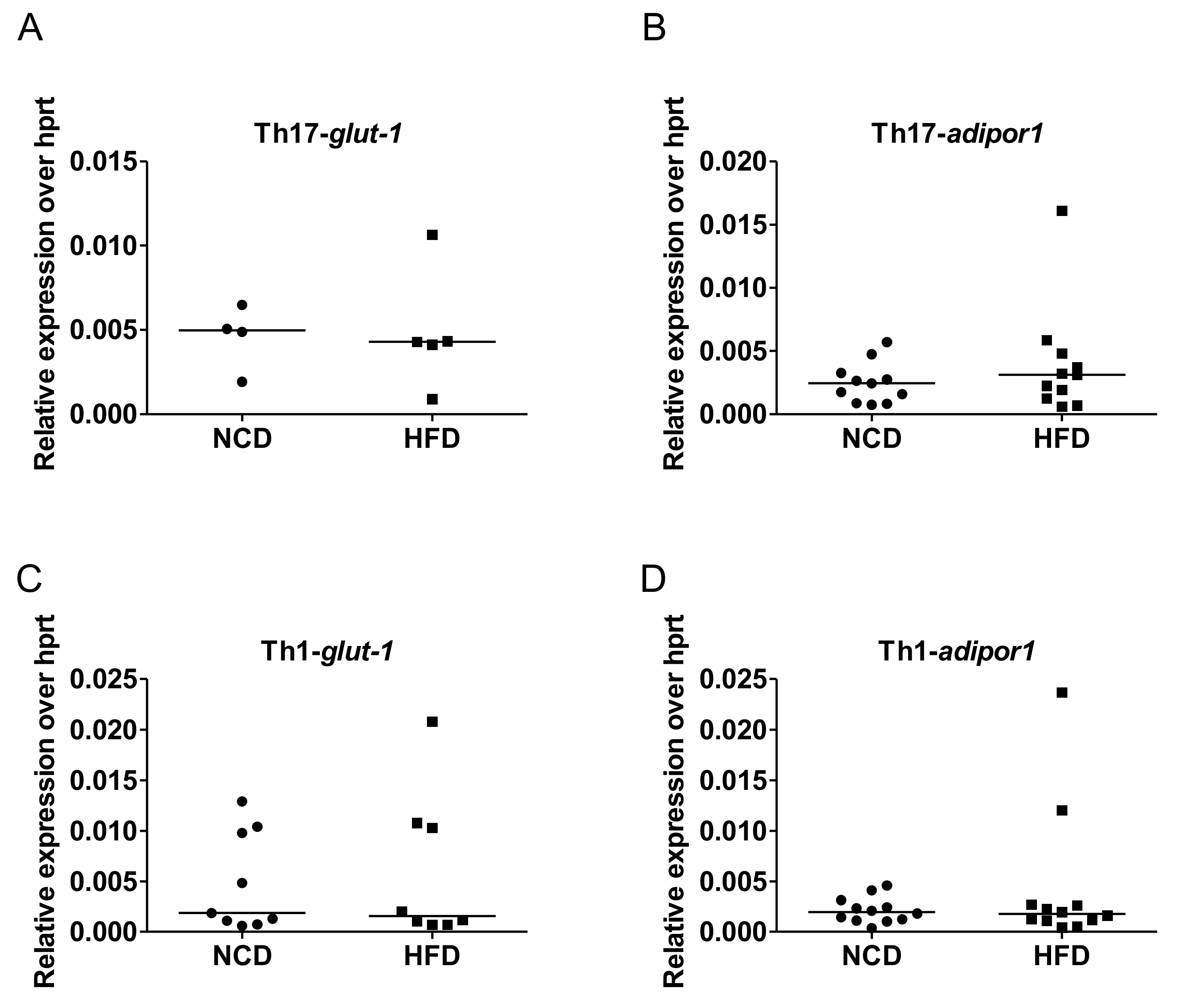

Supplement: Supplementary Figure 6 — Obesity does not influence adiponectin receptor or glucose transporter expression on Th1 and Th17 cells. (A–D) Relative mRNA expression of glucose transporter (glut-1) and adiponectin receptor 1 (adipor1) on purified Th17 (A,B) and Th1 cells (C,D) in normal control diet (NCD) and high fat diet (HFD) mice. Two-tailed non-parametric Mann–Whitney U-test was performed for statistical analysis. [file Image_6.TIFF]

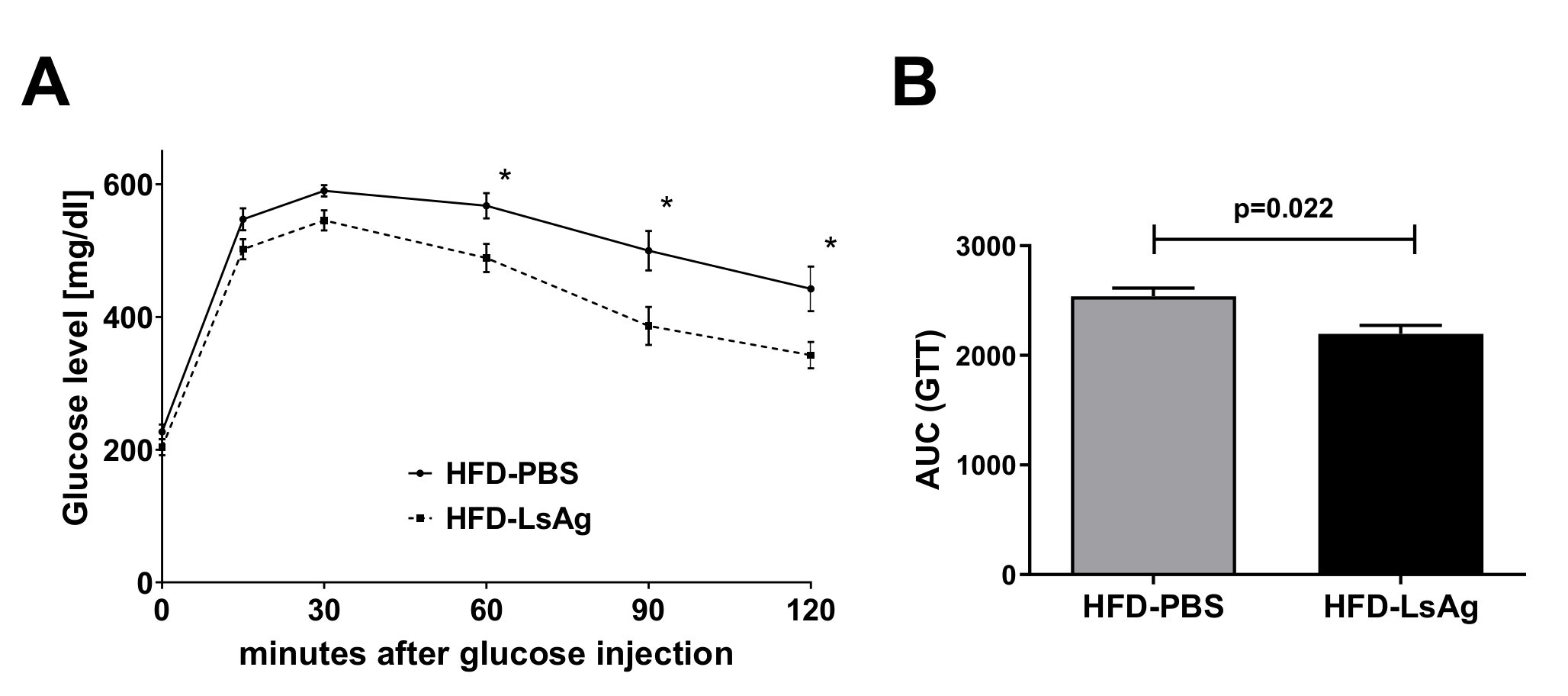

Supplement: Supplementary Figure 7 — LsAg improves glucose intolerance in obese mice. (A) Blood glucose concentrations over time after i.p. glucose challenge in PBS- and LsAg-administered high fat diet (HFD) mice. (B) Calculated area under curve (AUC) from the course of the glucose tolerance test. *p < 0.05. [file Image_7.TIFF]
